# Dynamic Conformational Change Regulates the Protein-DNA Recognition: An Investigation on Binding of a Y-Family Polymerase to Its Target DNA

**DOI:** 10.1371/journal.pcbi.1003804

**Published:** 2014-09-04

**Authors:** Xiakun Chu, Fei Liu, Brian A. Maxwell, Yong Wang, Zucai Suo, Haijun Wang, Wei Han, Jin Wang

**Affiliations:** 1 State Key Laboratory of Electroanalytical Chemistry, Changchun Institute of Applied Chemistry, Chinese Academy of Sciences, Changchun, Jilin, P.R. China; 2 College of Physics, Jilin University, Changchun, Jilin, P.R. China; 3 Department of Chemistry and Biochemistry, The Ohio State University, Columbus, Ohio, United States of America; 4 Department of Chemistry and Physics, State University of New York at Stony Brook, Stony Brook, New York, United States of America; University of Maryland, Baltimore, United States of America

## Abstract

Protein-DNA recognition is a central biological process that governs the life of cells. A protein will often undergo a conformational transition to form the functional complex with its target DNA. The protein conformational dynamics are expected to contribute to the stability and specificity of DNA recognition and therefore may control the functional activity of the protein-DNA complex. Understanding how the conformational dynamics influences the protein-DNA recognition is still challenging. Here, we developed a two-basin structure-based model to explore functional dynamics in *Sulfolobus solfataricus* DNA Y-family polymerase IV (DPO4) during its binding to DNA. With explicit consideration of non-specific and specific interactions between DPO4 and DNA, we found that DPO4-DNA recognition is comprised of first 3D diffusion, then a short-range adjustment sliding on DNA and finally specific binding. Interestingly, we found that DPO4 is under a conformational equilibrium between multiple states during the binding process and the distributions of the conformations vary at different binding stages. By modulating the strength of the electrostatic interactions, the flexibility of the linker, and the conformational dynamics in DPO4, we drew a clear picture on how DPO4 dynamically regulates the DNA recognition. We argue that the unique features of flexibility and conformational dynamics in DPO4-DNA recognition have direct implications for low-fidelity translesion DNA synthesis, most of which is found to be accomplished by the Y-family DNA polymerases. Our results help complete the description of the DNA synthesis process for the Y-family polymerases. Furthermore, the methods developed here can be widely applied for future investigations on how various proteins recognize and bind specific DNA substrates.

## Author Summary

Protein-DNA recognition is crucial for many key biological processes in cells. Protein often undergoes large-scale conformational change during DNA recognition. However, the physical and global understanding of flexible protein-DNA binding is still challenging. Here, we developed a theoretical approach to investigate binding of a Y-family DNA polymerase to its target DNA during the DNA synthesis process. The results of electrostatic-controlled multi-step DNA binding process accompanied with multi-state conformational transition of protein occurring throughout are in remarkable agreement with experiments. During the process of protein-DNA recognition, the flexibility is found to facilitate both the conformational transition of protein (intra-chain dynamics) and DNA binding (inter-chain dynamics) simultaneously. Therefore, we provided a quantitative description of protein-DNA binding mechanism that flexibility or conformational change regulates DNA recognition dynamically, leading to high efficiency and specificity of function for protein-DNA recognition.

## Introduction

Protein-DNA recognition is critical to the life of cells. The interactions between proteins and nucleic acids are prevalent in many vital processes including DNA synthesis, gene transcription, chromosome assembly and disassembly, etc. Evidence has been accumulating that protein-DNA recognition events are often accompanied by conformational changes that favor formation of the required functional complex [1], [2]. Disordered regions with highly charged residues are widely found in DNA-binding proteins [3] and are often responsible for the conformational changes in proteins during DNA recognition [4]. Such a flexible charged segment in a protein is inclined to form a non-specific complex with DNA through abundant electrostatic interactions and therefore facilitates DNA recognition by reducing the dimensionality of target search processes through sliding along the DNA contour [3], [5], [6]. Protein-DNA recognition is found to be accelerated by this “facilitated diffusion” phenomenon [7]–[11]. Consequently, the formation of a functional protein-DNA complex has to undergo a transformation from non-specific diffusion to specific transition. Currently, an increasing number of structures of DNA-binding proteins both in unbound and DNA-bound forms are being determined experimentally by X-ray crystallography. The illustration of the different structural representations of proteins with and without DNA binding has significantly improved our understanding of protein-DNA interactions [12]. Conformational changes or structural flexibility are found to provide many benefits for biomolecular recognition [13]–[17], including fast association/dissociation rates, large complementary binding interfaces, high binding specificity accompanied with moderate binding affinity, etc. The intrinsic disorder in DNA-binding proteins is recognized to increase mobility in order to fine-tune the binding affinity [18] and also to have a contribution to the stability and specificity of the DNA-protein complex [1], [4], [19]. However, there is still a lack of knowledge on the dynamics of DNA binding mechanisms, and more specifically, the role of the flexibility or conformational dynamics in the process of the formation and function realization of the DNA-protein complex.

DNA polymerases catalyze DNA replication with a stepwise mechanism, starting with the specific binding to DNA at the replication fork, followed by the incorporation of a nucleotide into the nascent DNA strand. Y-family polymerases are a group of specialized DNA polymerases, which have evolved to facilitate replication through various DNA lesions although they can replicate undamaged DNA with low-fidelity and poor processivity [20]. In spite of the fact that Y-family polymerases share little sequence identity with other high-fidelity DNA polymerases, numerous structural investigations have revealed that Y-family polymerases retain the conserved right-handed polymerase core architecture composed of a thumb (T), palm (P) and finger (F) domains common to all DNA polymerases [21]–[23]. In addition, Y-family polymerases possess a unique domain linked to the polymerase core, termed little finger (LF) domain [21], or polymerase-associated domain (PAD) [22]–[24]. The LF domain has been found to be the major factor in determining the unique biochemical properties of different Y-family polymerases [25] and contributes to the overall binding affinity to DNA [21], [22]. *Sulfolobus solfataricus* DNA polymerase IV (DPO4) is a model Y-family polymerase that has been widely studied from both structural and kinetic perspectives [26]–[28]. The apo-DPO4 and DNA-bound DPO4 exhibit very different structural conformations [26], implying that there is an “open to close” conformational change during DPO4-DNA recognition. Similar conformational changes have also been inferred from crystal structures of several human Y-family DNA polymerases [23], [29]–[32]. Investigations of DNA polymerization have mainly focused on the stages of the catalytic process related to the nucleotide incorporation [33]–[40], which is the central step in DNA synthesis. However, a recent stopped-flow FRET investigation has suggested that DNA recognition and binding is a complex, multi-step process in which conformational change steps may occur separately from complete formation of the specific DPO4-DNA complex [41]. Nevertheless, a clear description for the initial recognition of the target DNA primer-template site by a DNA polymerase and the involved conformational dynamics is lacking. Previous results by experiments and simulation have given strong evidence that the highly flexible linker that connects the T and LF domains plays a major role in this conformational transition in DPO4 [26], [28], [42]. How the binding happens, as well as how the conformational dynamics and flexible linker participate in DPO4-DNA recognition, remain unclear.

Here, we performed thermodynamic and kinetic simulations to explore the conformational transitions of DPO4 during binding to the target site in DNA by developing a structure-based model (SBM). Certain types of SBM, satisfying the principle of minimally frustrated energy landscapes [43], [44], have been used to study biomolecular folding and binding transitions toward formation of the unique native structure [45]–[49]. Based on our previous work [50], [51], we extended the plain SBM to two-basin SBM with electrostatic interactions described by the Debye-Hcükel model, aiming to capture the conformational dynamics of DPO4 during DNA recognition. Our results clearly showed that DPO4-DNA recognition involves 3D diffusion, then a short-range adjustment sliding on DNA and finally specific binding. The conformational changes in DPO4 happen throughout the binding process, with different stages representing different conformational distributions. The conformational dynamics in DPO4 seem to be fine-tuned by DNA. Meanwhile the different conformations of DPO4 have different effects on the DNA binding kinetics. We also found that electrostatic interactions on one hand facilitate the 3D diffusion as “steering forces” [15], [52]–[54], and on the other hand hinder short-range sliding on DNA due to formation of non-native kinetic traps. The efficiency of specific DPO4-DNA recognition is determined by the interplay between multiple binding stages. In particular, we point out that the flexibility of the positively charged linker not only contributes to the conformational distribution in DPO4, but is also responsible for the stabilization of the non-specific complex, and is therefore critical for DPO4-DNA recognition. Our methods, with explicit consideration of the non-specific and specific interactions between DPO4 and DNA, provide a detailed description of the process of DPO4 binding to its target DNA. This work illustrates the process of specific protein-DNA recognition and the accompanying conformational dynamics, and thus enriches our understanding of the catalytic mechanism of DNA polymerization.

## Results

### DPO4 binds to DNA dynamically

**Figure 1:**
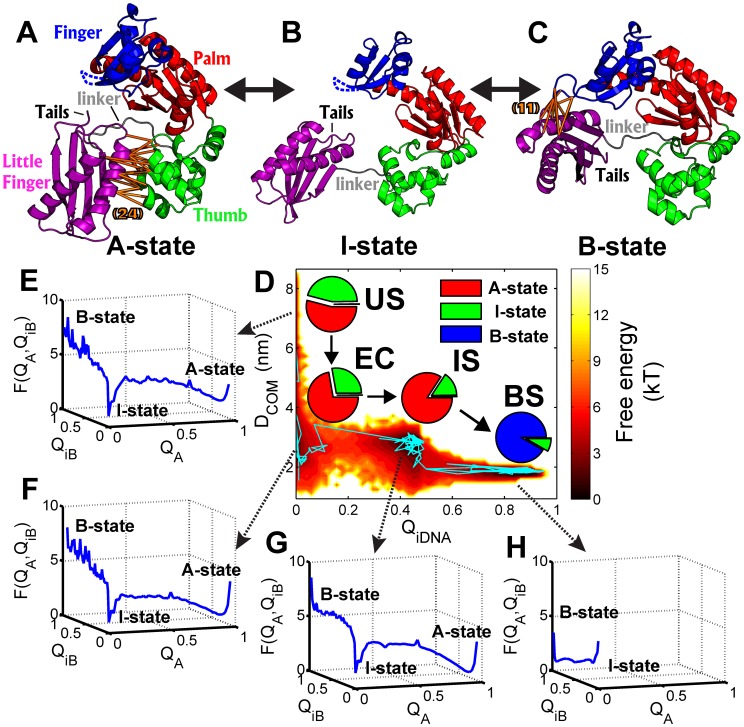
Structural representation of DPO4 and free energy landscapes. The structure of the A-, I- and B-states of DPO4 are shown in (A), (B) and (C), colored by: the F domain, blue; the P domain, red; the T domain, green; the LF domain, purple; the linker between the LF and T domain, grey. The specific interactions formed by the LF domain with the T domain in the A-state and the F domain in the B-state are drawn by orange lines in (A) and (C), and the number of the specific interactions are indicated in parentheses, respectively. The unstructured loop in the F domain is shown by the blue broken lines in (A) and (B). (D) The binding free energy is shown as a function of *Q_iDNA_* and *D_COM_* at 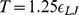. *Q_iDNA_* describes the native similarity of binding of DPO4 to DNA, *D_COM_* describes the distance between DPO4 and DNA. The four free energy minima correspond to different binding stages: the US, EC, IS and BS. A constant temperature simulation at 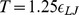 was performed and the trajectory is shown to validate the continuity of the adjacent stages in free energy landscapes. (E–H) The free energy landscapes of conformational dynamics in DPO4 are shown as a function of *Q_A_* and *Q_iB_* at each binding stage. *Q_A_*, *Q_iB_* measures the native similarity of the LF domain interacting with the T domain in the A-state and the F domain in the B-state, respectively. The A-, I- and B-states of DPO4 can be observed in the US, EC and IS, with different population distributions at different binding stages. Only the I- and B-state of DPO4 can be observed in the BS. The populations of the three states of DPO4 at each binding state are plotted as pie-charts in (D) near the corresponding free energy minima.

Without binding to DNA, the crystal structure of DPO4 is present as an “Apo” state (A-state) (Figure 1A), which is quite different from the conformation observed in the DNA-Bound state (B-state) (Figure 1C) [21], [26]. The major difference in the A- and B-state of DPO4 is the location and orientation of the LF domain relative to the F, P and T domains and folding of a disordered region in the F domain, while the other individual domains do not show significant conformational changes. Therefore, DPO4 undergoes a typical “A to B” conformational transition coupled with folding of the disordered loop in the F domain during binding to DNA.

To investigate the binding of DPO4 to DNA, we plotted the 2D free energy landscapes along *Q_iDNA_* and *D_COM_* sampled by Replica Exchange Molecular Dynamics (REMD) [55] (Figure 1D). *Q_iDNA_* is the fraction of native contacts between DPO4 and DNA, and *D_COM_* is the distance of the center of mass between DPO4 and DNA. *Q_iDNA_* and *D_COM_* are binding reaction coordinates, which monitor the degree of binding process. The free energy landscapes combining with additional constant temperature simulation trajectory showed that the DPO4-DNA binding proceeds with four kinetically connected stages, including the stage with unbinding states (US), the stage with encounter complex (EC), the stage with intermediate states(IS) and the stage with binding states (BS), corresponding to typical biomolecular recognition [56]. In detail, we found that DPO4 undergoes conformational dynamics at each stage during DNA recognition from the free energy landscapes along *Q_A_* and *Q_iB_* (Figure 1E–H), where *Q_A_* and *Q_iB_* are the fractions of the native inter-domain contacts of the LF domain in the A- and B-state of DPO4, respectively. There are three free energy minima for the conformational dynamics of DPO4, corresponding to the state with inter-domain interactions forming between the LF and T domain (A-state, *Q_A_*>0.8, *Q_iB_* = 0), the state with inter-domain interactions forming between the LF and F domain (B-state, *Q_A_* = 0, *Q_iB_*>0.8), and an intermediate state, in which the LF domain does not interact with the F or T domain (I-state, Figure 1B, *Q_A_*<0.1, *Q_iB_*<0.1).

By investigating the folding of the flexible loop in the F domain, we found that the “disorder to order” transition of the loop can be observed during DNA recognition (Figure S6A in Text S1). The loop at the unbound state is shown to be under a fast dynamic transition of the conformational circulation including a wide range of abundant disordered structures due to the small free energy barrier between them (Figure S6B in Text S1). This observation that the disordered region is not completely but dynamically disordered has been widely seen in the investigations of Intrinsically Disordered Proteins (IDPs) [57]–[60]. It is expected to benefit IDPs for high binding specificity but moderate affinity and fast kinetics [61]. We found that the degree of binding of the LF domain to the F domain is strongly coupled to the ordering of the loop in the F domain (Figure S6A in Text S1) and can be used to measure the extent of the formation of order in this loop.

The free energy landscapes of the conformational dynamics of DPO4 gave strong evidence that this “A to B” transition in DPO4 occurs in the sequence by “A–>I–>B” through an inevitable I-state during the binding of DPO4 to DNA (Figure 1E–H). We found that at each binding stage (US, EC, IS or BS), DPO4 is in conformational equilibrium among the A-, I- and B-state. The population distribution of the three states of DPO4 is different at different binding stages. At the simulation temperature 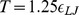 (temperature is in energy unit by multiplying Boltzmann constant *k* and 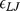 is the strength of the long-range Lennard-Jones potential), in the US the A-state occupies the highest population of 54.1%, the I-state occupies a slightly lower population of 45.7% and the B-state occupies only a small population of 0.2%. As the binding proceeds through the EC and IS stages, the population of the A-state increases and the I-state decreases while the population of the B-state remains small. However, at the last transition stage of the IS to the BS, the populations of the three states of DPO4 change significantly, showing that only the I-state and the B-state can be observed and the B-state is dominant. Overall, the free energy landscapes showed that DPO4-DNA recognition is a multi-step binding process through four distinct binding stages (the US, EC, IS and BS) accompanied with multi-state conformational transition among three different states of DPO4 (the A-, I- and B-states) occurring throughout. In DPO4-DNA recognition, the four binding stages describe the inter-chain dynamics for DNA binding process, while the three states describe the intra-chain dynamics for conformational transition in DPO4. These results provide a dynamic picture of DPO4-DNA recognition with the changes in the inter-chain interactions and adjustments in the intra-chain conformations, simultaneously.

### Temperature regulates the conformational dynamics of DPO4 during DNA binding

**Figure 2:**
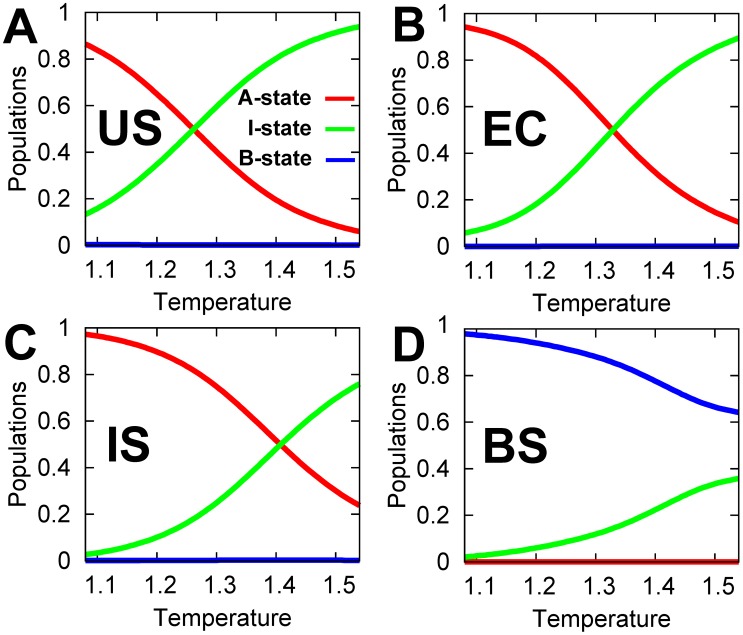
Conformational dynamics of DPO4 change with temperature. The population distributions of the A- (red lines), I- (green lines) and B-state (blue lines) in the (A) US, (B) EC, (C) IS and (D) BS are shown as a function of temperature. Temperature is energy unit (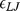).

To further investigate the conformational dynamics of DPO4 during DNA recognition we performed temperature dependency studies. For the US, EC and IS, increasing temperature leads to decreasing population of the A-state, increasing population of the I-state and almost unchanged population of the B-state which is close to 0; while for the BS, increasing temperature leads to decreasing population of the B-state, increasing population of the I-state and almost unchanged population of the A-state which is close to 0 (Figure 2). The results indicated that the A- and B-state of DPO4 are enthalpy-driven and favored at low temperatures, while I-state of DPO4 is entropy-driven and favored at high temperatures. It is worth noting that the temperature here cannot be exactly the same in value as the temperatures at which DPO4 is typically studied experimentally due to the coarse-grained feature of our model. DPO4 has been found to function *in vivo* at extreme conditions of 80°C and PH 2 to 3 [62]. Our previous folding/unfolding simulations inferred that at the functional temperature, the conformation of DPO4 has a tendency to change at the interface of the T and LF domain, as well as the linker region [28], leading to the formation of the I-state. Here, we found that the DPO4 performs DNA recognition with certain populations of DPO4 with the I-state structure in the US and that the population in the I-state increases significantly with increasing temperature. In the BS, DPO4 in the B-state is dominated over a wide range of temperatures with only small populations of DPO4 in the I-state. Therefore the activity of DPO4 is expected to be maintained with changing temperature. The results are consistent with the experimental findings that the mechanism of DPO4 is temperature independent [27], [35], [63]. In order to investigate the conformational dynamics of DNA recognition, we chose the temperature (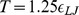), at which the populations of the A- and I-state of DPO4 in the US are similar while the B-state of DPO4 is dominated in the BS to guarantee catalytic efficiency. Due to the observation of DPO4 in the I-state, this temperature is expected to be higher than room temperature, at which DPO4 was experimentally identified as having A-state structure [26], but lower than the overall melting temperature, since we did not observe the global folding/unfolding transition of DPO4 during the simulations.

### DPO4 binding to DNA is a transition process from non-specific to specific recognition

In DPO4-DNA recognition, the EC is formed after the 3D diffusion, in which large translation entropy is lost. To achieve a free energy minima at the EC, there must be some stabilizing interactions between DPO4 and DNA [64]. However, there is little native interactions observed in the EC as shown in free energy landscapes with *Q_iDNA_*<0.1 (Figure 1D), implying that the stabilizing forces in the EC are mostly non-native. By investigating the DPO4-DNA inter-chain interaction energy during binding (Table 1), we found that there are many non-native electrostatic interactions in the EC while the native interactions are almost unformed. It should be noted that the non-native Lennard-Jones (LJ) interactions are represented only by an exclusive volume repulsive term, which does not contribute to the stabilization of the EC (See details in “**Materials and Methods**” and Text S1). The EC is therefore stabilized by abundant non-native electrostatic interactions. The inter-chain electrostatic interactions have been regarded as the “steering forces” to facilitate the biomolecular recognition at the first stage and then stabilize the temporary partial binding EC to compensate the entropy lost [15], [52]–[54]. Therefore, transition from the US to the EC is facilitated by non-native electrostatic interactions and is expected to be non-specific. The 3D diffusion can be regarded as non-specific binding.

**Table 1:** Protein-DNA interaction energy in each binding state at 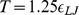.

Stage	US		EC		IS		BS	
Energy Part	Native	Non-native	Native	Non-native	Native	Non-native	Native	Non-native
*E_Elect_* *	−0.01±0.03	−0.12±0.69	−0.55±0.60	−15.10±4.27	−10.68±0.82	−11.17±1.11	−10.43±0.80	−12.08±1.22
*E_LJ_* #	−0.00±0.03	0.01±0.11	−0.29±0.56	1.26±1.31	−18.43±2.56	0.23±0.30	−42.01±4.43	0.44±0.38

*Energy is in the unit of 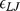.

# In structure-based model, the non-native LJ potential is represented by the volume repulsive term in Hamiltonian. See details in “**Materials and Methods**.”

**Figure 3:**
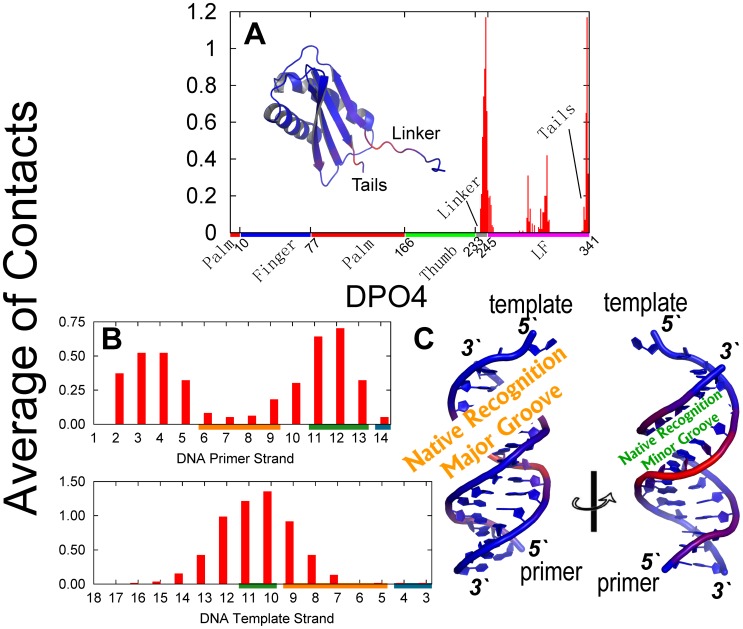
The contacts between DPO4 and DNA at the EC. Contact is based on a cut-off algorithm (See details in Text S1). (A) Average of the contacts of each residue in DPO4 and a contact-colored structure of DPO4 are shown. Since there are no contacts formed at the polymerase core, only the LF domain and the linker are shown. The color on the x-axis represents the different domains or regions in DPO4, the coloring scheme is same as that in Figure 1A. (B) Average contacts of each nucleotide in DNA are shown by primer and template strand, separately. The color on the x-axis represents the different regions of the DNA duplex: orange, the native recognition major groove; olive, the native recognition minor groove; dark cyan, the native recognition region at terminal of DNA. The native contacts are shown in Figure 9. (C) The DNA is colored by the average contacts of sugar, base and phosphate group. The color in the structure in (A) and (C) from blue to red corresponds to the number of contacts from zero to the largest.

The findings that the EC is stabilized by non-native electrostatic interactions are consistent with previous structural analysis [19], [65], [66]. Here, we introduced a cut-off algorithm which can take consideration of the non-native interactions to investigate the non-native contacts in the EC. For DPO4, the non-native contacts are entirely formed at the LF domain and the linker region, while the other domains seem to be away from DNA (Figure 3A and Figures S7 and S8 in Text S1). Specifically, the regions with strongest interactions in DPO4 are located at the linker and the tails of the LF domain. Both of these regions are very flexible, leading to a large capture radius. In addition to many positively charged residues present at these two regions, the protein-DNA binding is expected to be facilitated by the “fly-casting” mechanism through the flexibility and electrostatic interactions [13], [15], [16]. Our simulation results confirm that the LF domain and the linker in DPO4 are the recognition regions for DPO4-DNA binding as observed experimentally [21], [22]. For the primer/template DNA, there are more contacts located at one side of the double-stranded duplex, comprised of the native recognition minor groove and the non-native major groove, than the other side, comprised of the native recognition major groove. This implies that DPO4 moves selectively along DNA in the EC. In particular, although DPO4 adopts three different conformations in the EC, the contact maps for the three states are quite similar and the energy values of the interactions are close to each other (Table S1, Figures S7 and S8 in Text S1). It is worth noting that DPO4 in the B-state shares a slightly wider and stronger distribution of non-native contacts with DNA, implying that DPO4 interacts more strongly with DNA in the B-state than in the A- and I-states. However, there is a very small population of DPO4 in the B-state at this stage, and so the effect is expected to be minimal.

**Figure 4:**
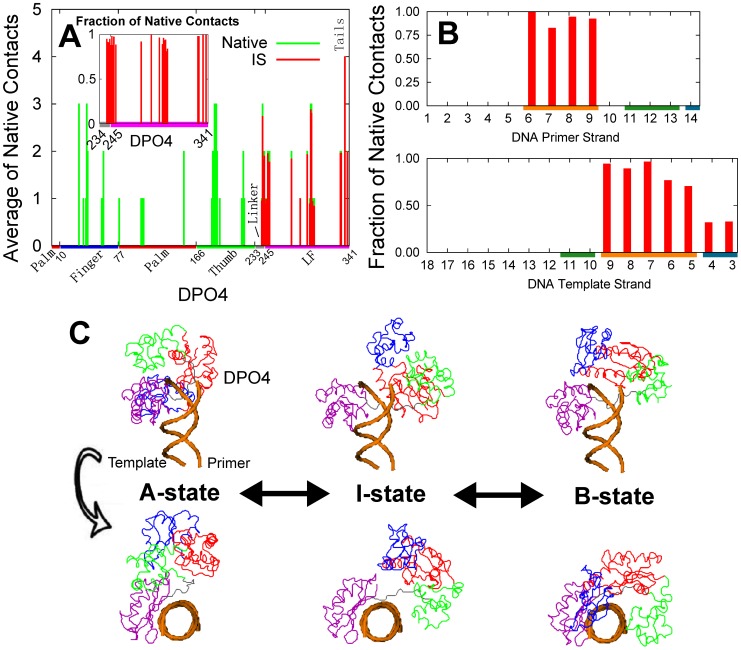
Native contacts between DPO4 and DNA as well as the structural illustrations of DPO4-DNA complex in the IS. (A) The average of inter-chain native contacts for each residue in DPO4 are shown. The fraction of native contacts, which measures the degree of the formation of the native contacts, is shown in the insert figure. (B) The fraction of native contacts is shown for each nucleotide of either the primer or the template strand. The color on the x-axis in (A) and (B) is the same as that in Figure 3. (C) Typical structures of the A-, I- and B-state of DPO4 binding with DNA in the IS are shown. The coloring strategy for DPO4 is the same as that in Figure 1A.

After the non-specific EC, the IS is formed through specific binding by both electrostatic and non-electrostatic native contacts (Table 1). By investigating the native contacts in the IS (Figure 4), we found that the LF domain and linker in DPO4 form almost all native contacts with DNA while the F, P and T domains form very few native contacts. Consequently, the native contacts in the IS are very similar in the three states of DPO4 (Figure S9 and S10 in Text S1), implying that the conformational dynamics of DPO4 has little influence on the interactions with DNA in this stage of binding. Our results indicated that in the IS, the LF domain and linker in DPO4 are already in the native binding state, and then the last binding stage seems to involve binding of the other domains of DPO4 to the target sites on DNA. On the other hand, the native contacts at the major groove of the DNA duplex are almost formed. Meanwhile, half of the native contacts at the terminal of the DNA template strand are formed while there are no native contacts formed at the minor groove of the DNA duplex. This is consistent with the fact that the major groove of DNA is usually the primary binding site [67], [68]. It is worth noting that the electrostatic interactions, including native and non-native ones are very similar in the IS and BS, while the major difference is the formation of the native LJ contacts, of which the energy shows a remarkable increase in the BS. This implies that DPO4 finishes the formation of all native electrostatic interactions in the IS, and the last evolving step from the IS to the BS is non-electrostatic, specific binding and is expected to be electrostatic independent.

In summary, the process of DPO4-DNA binding is quite different at different stages and includes a switch from non-specific to specific binding. First, DPO4 undergoes 3D diffusion steered by the long-range attractive electrostatic interactions between DNA and the LF domain as well as the linker of DPO4 to form the non-specific EC with DNA; then a transition to the IS evolves through specific electrostatic and LJ interactions between DNA and the LF domain as well as the linker of DPO4; finally, DPO4 forms the native binding with DNA through specific LJ interactions including the binding of DNA to the F, P, and T domains.

### Electrostatic interactions through salt concentrations modulate the binding kinetics step by step

**Figure 5:**
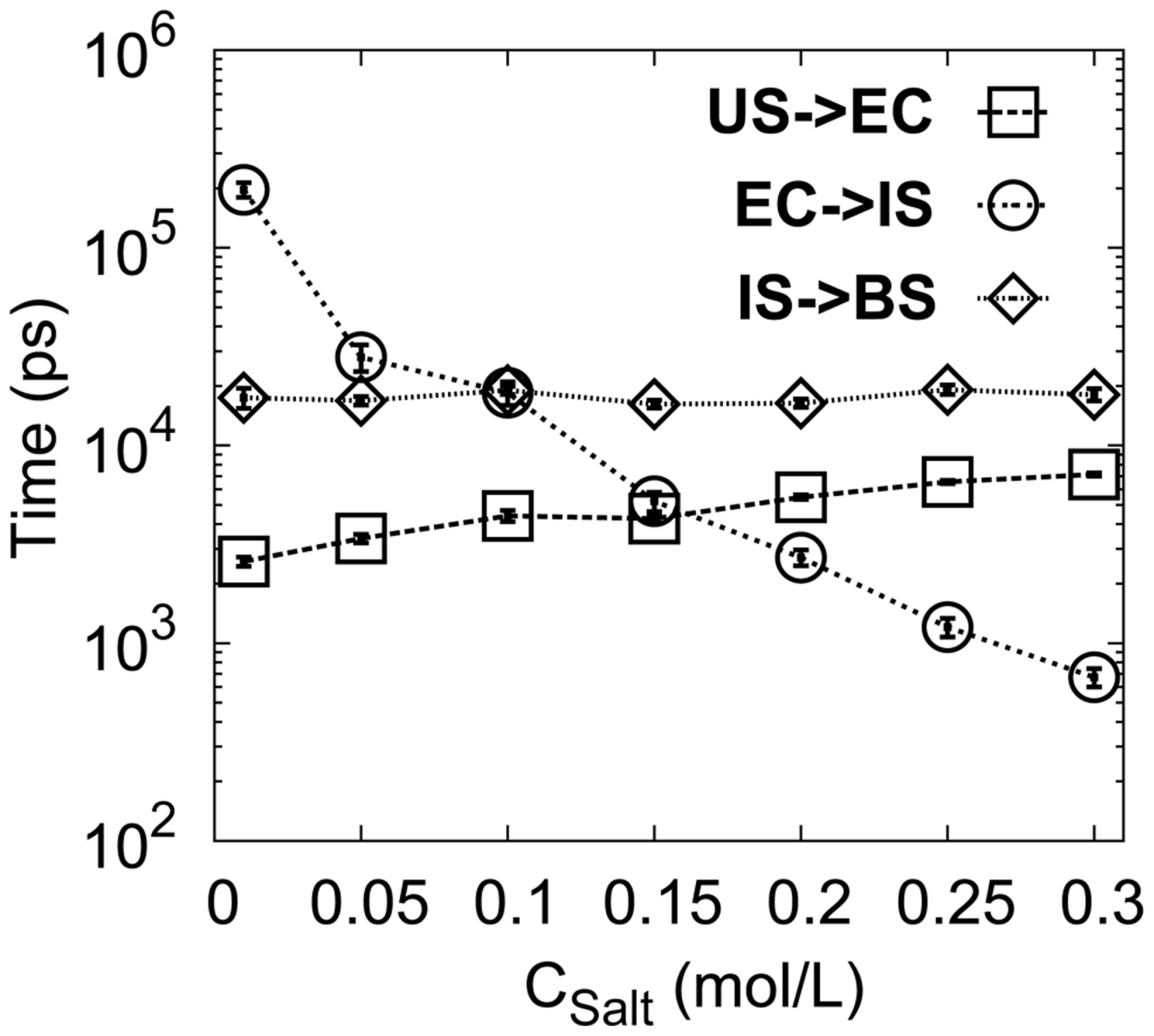
MPT as a function of *C_Salt_*. *C_Salt_* is the salt concentration. The error bar represents the standard error of the corresponding MPT.

The thermodynamic results imply that electrostatic interactions play a very important role in DPO4-DNA recognition process including stabilizing the EC with non-specific interactions and the evolution to the IS from the EC with specific interactions. To investigate the role of electrostatic interactions in the kinetics of the recognition, we analyzed the Mean Passage Time (MPT) at different salt concentrations, corresponding to different strengths of electrostatic interactions (Figure 5). In Debye-Hcükel model, the effect of salt concentration is modulated by the length of Debye radius. With increasing salt concentration, the screening effect of the implicit ion increases, leading to a decrease in the strength of electrostatic interactions.

With decreasing salt concentration (increasing electrostatic interactions), we found that the rate of 3D diffusion from the US to the EC increases by up to 3-fold, implying that the electrostatic interactions facilitate binding at an early stage. This is consistent with the thermodynamic analysis and the electrostatic interactions are suggested to be the “steering forces” in biomolecular recognition [15], [52]–[54]. Intriguingly, we found that increasing the electrostatic interactions disfavors the transition from the EC to the IS, as decreasing salt concentration led to slower kinetics by greater than two orders of magnitude. Since the transition from the EC to the IS involves forming specific electrostatic interactions, the negative correlation between the binding rate and the strength of electrostatic interactions was surprising. From our thermodynamic analysis, we found that there is a wide distribution of non-native electrostatic interactions formed in the EC, due to the charged characteristic of the DPO4-DNA system. The non-native interactions reduce the dimensionality for DPO4 binding from 3D in the US to 1D in the EC, leading to efficient search for the specific target site on DNA. However, these intermittent, transient, non-native interactions can lead to kinetic traps on energy landscape to slow down the binding. Recently, Marcovitz and Levy investigated the interplay between the non-specific and specific binding modes of protein-DNA recognition taking into consideration the structures of proteins and DNA [69], [70]. They proposed that there is a moderate degree of frustration between the two binding modes. This frustration, which regulates protein-DNA recognition, is found to be correlated with the overlap between the non-specific and specific binding patches. Low frustration will lead to a rugged energy landscape for non-specific binding but rapid transition from non-specific to specific binding. In contrast, high frustration will result in a smooth landscape for sliding but a high free energy barrier for non-specific to specific binding [69], [70]. In reality, the degree of frustration in protein-DNA binding is supposed to be optimized to satisfy the biological function. In DPO4-DNA recognition, transition from the non-specific EC to the specific IS involves switching the electrostatic interactions from non-specific to specific ones, leading to frustration or free energy barrier. Increasing electrostatic interactions will increase this frustration between the two binding modes, resulting in decreasing recognition rate. At the last stage, the kinetic rate was independent of salt concentration, which is consistent with thermodynamic analysis that the transition from the IS to the BS does not involve electrostatic interactions.

At low salt concentration, the transition from the EC to the IS is the rate-limiting step in DPO4-DNA recognition. With increasing salt concentration, the rate of DPO4 adjusting on DNA increases and the last transition stage from the IS to the BS becomes the rate-limiting step. Therefore, the efficiency of DPO4-DNA recognition cannot be accelerated by electrostatic interactions, especially in the case of low salt concentrations, at which the binding kinetics will be decelerated by the charged interactions, due to the very stable non-specific EC.

### Flexibility in linker facilitates the recognition by increasing the efficiency of non-specific to specific binding

During the conformational transition from the A- to I-state in DPO4, the linker region between the T and LF domain changes significantly, implying that the linker is very flexible [26], [42]. The flexibility of the linker has been confirmed by heat denaturization experiments [27]. From our thermodynamic analysis, we found that the linker is an important part of the initial recognition region in DPO4-DNA binding in the EC and is the stabilizing segment in the IS. Thus it is important to investigate the role of the flexibility of the linker during DNA recognition. In our SBM, the conformation is attracted toward the native structure, corresponding to the basin of the energy landscapes. Since there are two basins at the bottom of the energy funnel, we performed two groups of simulations, in which the conformations of the linker are attracted toward the A-state and B-state structure separately, with different scaling parameters 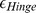. 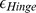 controls the rigidity of the linker biased to the A-state or B-state in our model and a small (large) value of 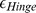 corresponds to low (high) rigidity. (Details in “**Materials and Methods**”).

**Figure 6:**
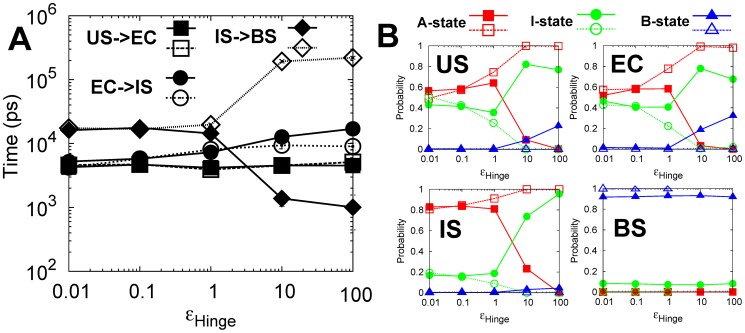
MPT and the conformational dynamics of DPO4 change with the flexibility of the linker. 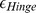
 is the parameter which controls the flexibility of the linker. There are two groups of 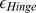 shown. The filled symbols correspond to the linker biased to the B-state while the empty symbols correspond to the linker biased to the A-state. (A) MPT as a function of 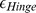. (B) The conformational population distribution of the A-, I- and B-state of DPO4 in the US, EC, IS and BS with different 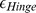. Notice that when 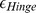 equals 10.0 and 100.0, the BS cannot be observed in the simulations when the linker is biased to the A-state. MPT is calculated as the largest observation time. The error bar represents the standard error of the corresponding MPT.

The flexibility in the linker and biasing to either A- or B-state has little effect at the step of 3D diffusion from the US to the EC (Figure 6A). As binding proceeds, we found that the transition from the EC to the IS is accelerated by the flexibility of the linker. This could be due to the fact that the flexible linker escapes from the non-native interactions traps more easily than the rigid one when DPO4 performs the short-range adjustment sliding on DNA. Notably, with low flexibility (high 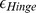), the linker biased to the B-state shows slower binding kinetics than when biased to the A-state. In other words, transition from the EC to the IS disfavors the B-state of the linker. The results can be explained by the contact map in the EC (Figure S7 and S8 in Text S1), in which DPO4 in the B-state has wider and stronger non-native electrostatic interactions with DNA than in the A- and I-states. Biasing the linker to the B-state increases the relative population of DPO4 in the B-state (Figure 6B), leading to more stable EC trapping. Therefore, to achieve a high efficiency of sliding along a DNA backbone, a flexible rather than rigid linker in DPO4 is required.

Biasing the linker to the B-state or A-state is found to significantly favor or disfavor the last binding stage, respectively (Figure 6A). The binding from the IS to the BS corresponds to the specific binding of the F, T and P domain of DPO4 to the target site on DNA accompanied with the formation of the B-state of DPO4. Although the three states in DPO4 show no differences in the inter-chain interactions with DNA in the IS (Figure S9 and S10 in Text S1), strongly biasing linker to the B-state facilitates the “A to B” conformational transition in DPO4 (Figure 6B), and is expected to accelerate the rate from the IS to the BS. Additionally, with the linker strongly biased toward the B-state, the rate-limiting step is changed to the transition from the EC to the IS, corresponding to the transformation process from non-specific to specific binding.

### DPO4 dynamics regulates recognition kinetics

**Figure 7:**
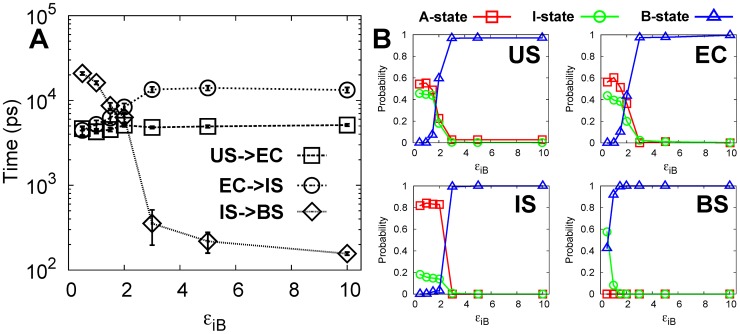
MPT changes with the conformational dynamics. 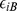
 is the strength of the specific native contacts between the LF and F domain in B-state of DPO4. 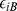 controls the conformational dynamics of DPO4. (A) MPT as a function of 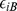. (B) The conformational distribution of the A-, I- and B-state of DPO4 in the US, EC, IS and BS with different 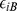. The error bar represents the standard error of the corresponding MPT.

During DNA binding, DPO4 is found to be under the conformational equilibrium between the A-, I- and B-states. To investigate how these conformational dynamics in DPO4 influences DNA binding, we scaled the strength of the biasing toward the B-state structure in our SBM and calculated the binding kinetics (Figure 7A). 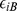 is the scaling parameter, which controls the strength of the specific interactions in the B-state of DPO4. A large (small) 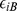 corresponds to strong (weak) specific interactions in the B-state of DPO4. Increasing 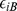 leads to more populated B-state of DPO4 and the population of the A- and I-state of DPO4 decreases accordingly (Figure 7B).

Interestingly, the conformational dynamics has little effect on the 3D diffusion of the US to the EC as the three states of DPO4 anchor DNA molecules with similar rates (Figure 7A). For the transition form the EC to the IS, the rate was found to be decelerated by increasing 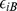. This is due to the fact that increasing 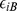 leads to a higher population of the B-state of DPO4 in the EC and the strong interactions of the B-state of DPO4 with DNA slow the short-range adjusting kinetics by forming kinetic traps. Finally, the B-state of DPO4 facilitates the transition of the IS to the BS as 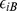 increases. The rate-liming step is changed depending on the value of 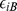. At small 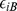, DPO4-DNA binding is limited by the last specific transition from the IS to the BS. With higher 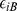, the rate of the IS to the BS transition increases while the rate of the transition from the EC to the IS decreases and becomes rate-limiting. The optimal case corresponds to 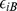 between 1.5 and 2.0, at which the transition rates from the EC to the IS and the IS to the BS are similar and the overall binding rate is the fastest.

## Discussion

Using circular dichroism spectroscopy and fluorescence-based thermal scanning experimental technology, Sherrer et al. observed an unfolding intermediate as temperature was increased from 26 to 119°C [27]. In our DPO4-DNA simulations here we detected the I-state of DPO4 during DNA recognition in addition to the A- and B-state of DPO4. The structural characteristic of the I-state of DPO4, which has a flexible and extended linker and four well-folded domains (excepted the unstructured loop in the F domain), is in accord with the unfolding intermediate detected through both experimental measurements and our previous simulation investigation on the folding and unfolding of DPO4 [27], [28].

### The binding influences on conformational changes

Our analysis showed that DPO4 is under conformational equilibrium between the A-, I- and B-states during DNA binding process, with different distributions of populations at each stage. Since the contact map analysis showed the inter-chain interactions of the three states of DPO4 with DNA are very similar, the effect of DNA binding is expected to modulate the conformational equilibrium of DPO4 through entropy. As binding proceeds, the conformational search space for the conformational dynamics of DPO4 is significantly reduced by the spatial limitation, leading to the fact that binding favors the low-enthalpy and low-entropy A-state or B-state, rather than high-enthalpy and high-entropy I-state. In detail, as binding proceeds before the final BS, the populations of the A- and I-state increase and decrease, respectively; in the BS, the B-state is dominated with a moderate population of the I-state. Structurally, the interactions between the LF and T domains in the A-state of DPO4 block the DNA-binding cleft, thereby preventing the formation of the A-state in the BS. Therefore, transition of the IS to the BS requires unraveling of the contacts between the LF and T domain in the A-state of DPO4, which is a time consuming process. At moderate salt concentrations, we found that the last step consisting of specific binding of DPO4 to DNA coupled with conformational transition to the B-state of DPO4 is the rate-limiting step in DPO4-DNA recognition. Compared with the B-state of DPO4, the I-state of DPO4 in the BS has weaker inter-chain interactions with DNA (Table S1 in Text S1) and more extended conformation, leading to a larger solvent-accessible area at the DNA binding site. Therefore, the conformational equilibrium of DPO4 being in the BS will lead to fluctuated interactions between DPO4 and DNA substrate, which may contribute to the ability of DPO4 to accommodate the bypass of various DNA lesions [71]–[74], while contributing to the low fidelity of DNA synthesis typical in Y-family DNA polymerases. It would be interesting to investigate how the presence of various lesions in the DNA substrate might affect the DPO4-DNA recognition process.

### Flexibility of the linker

The flexibility of the linker, which promoted the unfolding intermediate state during the unfolding experiments [27], is demonstrated to play a very important role in the distribution of the conformational states in DPO4 in our simulations. Additionally, the flexible linker with many positively charged residues, is found to be the “molecular recognition element (MoRE)” in the DPO4-DNA binding and therefore controls the efficiency of the recognition. In our previous simulations and experiments, flexible linkers in multi-domain proteins have been found to facilitate DNA recognition through diffusion in reduced dimension [5], [75]. Based on the contact map analysis, we found that the flexible linker forms many non-native electrostatic interactions in the EC. This indicates that the linker, especially the MoRE, is responsible for switching of the binding modes from 3D diffusion to the short-range adjustment sliding on DNA, which corresponds to the “facilitated diffusion” [7]–[11]. Furthermore, we found that the transition from the EC to the IS is accelerated as the linker becomes more flexible. The non-specific EC is expected to be dynamically ordered due to the high flexibility of the linker, leading to the “fuzzy complexes” [76], [77]. The fluctuating form of the EC with weak strength of interactions allows for rapid searching near replication fork to allow DPO4 to find the targeted primer-template site to achieve recognition adaptability [4], [19].

### Experiments of DPO4 binding to DNA

**Figure 8:**
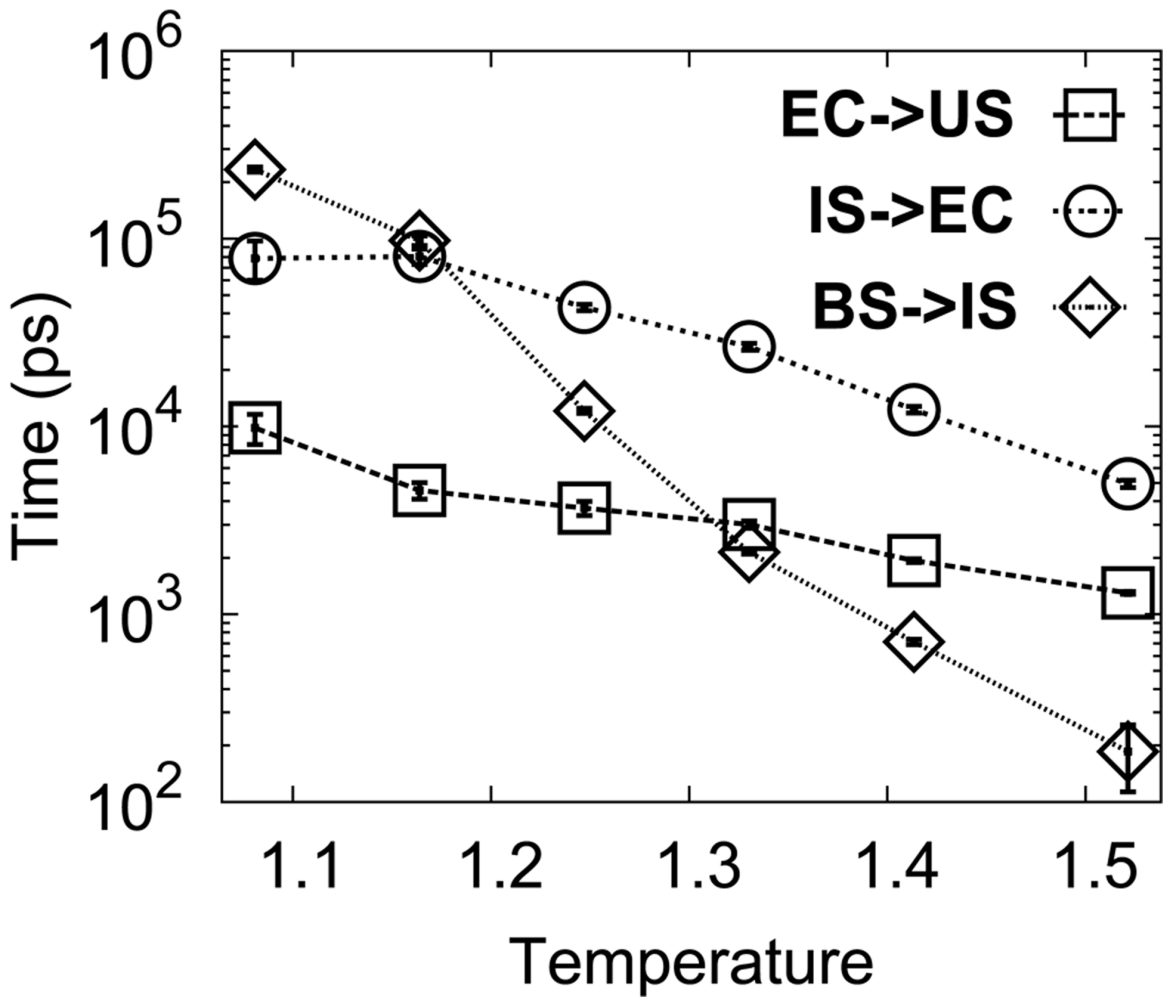
MPT of dissociation of DPO4-DNA binary complex as a function of temperature. The error bar represents the standard error of the corresponding MPT. Temperature is in energy unit (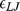).

Conflicting experimental evidence from a variety of technique [36], [39], [41], [78], [79] has indicated that the process of the association and dissociation of the DPO4-DNA complex is complicated, and a clear mechanism for DNA binding has remained elusive. Our results here are able to provide new insight into this mechanism and can help to connect the experimental results from different studies [36], [78], [79]. Initial ^32^P-based kinetic analysis has indicated that the release of DNA from DPO4-DNA complex is rate-limiting in steady-state catalysis with an observed dissociation rate of 0.02 *s* ^−1^ [78]. However, this value is three orders of magnitude slower than the dissociation rate measured by the fluorescence changes of a DPO4 Trp mutant, which was shown to have full catalytic activity [36]. Our results demonstrate that the DPO4-DNA recognition is a multi-state process and the abovementioned very different dissociation rates may come from different stages of releasing DNA. As shown in Figure 8, the rates of different dissociation stages vary significantly. At 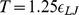, the transition from the BS to the IS proceeds faster than the transition from the specific complex IS to the non-specific complex EC. After the EC formation, DPO4 can easily release DNA with the fastest rate. Thus, the dissociation measured in steady-state kinetic analysis may correspond to the process of loss of the specific complex, including the transition from the BS to the IS and then the IS to the EC. In the fluorescence-based assays, fluorescence was monitored from T239W in the linker region. In our simulations we found that the linker region forms many non-native contacts with the DNA in the EC, where the polarity of the local environment of Trp-239 is different from that in the US. Therefore the dissociation rate measured by this fluorescence approach likely describes the transition from the EC to the US, which is demonstrated here to be much faster than the transition from the BS to the EC. In addition, the steady-state kinetic analysis showed that as temperature increases from 37°C to 56°C, the dissociation rate of DNA increases from 0.02 *s* ^−1^ to 0.11 *s* ^−1^ [35]. This is consistent with our results in Figure 8 which show that dissociation rate of DNA from the specific BS and IS to the non-specific EC increases as temperature increases. Interestingly, a recent single-molecule FRET investigation [79] measured a rate of DPO4-DNA dissociation which is intermediate between the values determined by ^32^P-based kinetic methods [78] and the Trp fluorescence [36]. This could be the result of measuring the dissociation of a mixture of complexes in the EC, IS and BS stages. Notably in this single-molecule FRET study [79], the authors observed multiple FRET efficiencies for the DPO4-DNA binary complex which they have attributed to a pre-translocated complex corresponding to the binary complex observed in crystal structures, and a translocated complex where DPO4 has shifted its contacts with the DNA by one base pair along the DNA helix. However, in light of our studies, it is possible that the different FRET efficiency levels may be due in part to the different conformations of DPO4 in the A-, I- or B-state in the EC, IS or BS.

### Mechanism of binding: Induced fit versus conformational selection

Binding mechanisms have been long time debated issues in biomolecular recognition. A rigid binding model, referred to as “lock-and-key” mechanism was proposed by Fischer more than a century ago [80] and successfully explained the enzyme reaction catalyzed by a rigid complementary substrate. When taking into consideration the flexibility in biomolecular recognition, two additional proposals emerged: “induced-fit” [81] and “conformational selection” [82]–[87]. The two binding scenarios are distinguished by whether the conformational changes happen before or after binding. An induced-fit binding mechanism has been suggested for protein-DNA recognition, due to the long-range electrostatic interactions and the geometrical size of DNA [88], [89]. However, our recent stopped-flow FRET study with DPO4 shows that the conformational changes occur in a step distinct from association or dissociation of the specific DPO4-DNA complex and several alternative multi-step binding pathways were proposed [41]. The experiment provides the evidence for the possible coexistence of “induced-fit” and “conformational selection” binding mechanism, leading to the fact that the conformational change step may occur either before or after DNA binding. Our results here are able to further expand this proposed binding mechanism and confirm that DNA binding by DPO4 includes a mix of both “induced-fit” and “conformational selection” [90]. Our simulations show that there is a conformational equilibrium at the early stages of DPO4-DNA binding (i.e. in the US, EC and IS) followed by a functional “A to B” switch at the last stage from the IS to the BS. Based on the free energy landscapes and structural analysis, we found that the A-state DPO4 has to evolve to the I-state before finally adopting the B-state.

This extended conformational selection process was widely found in flexible biomolecular recognition, leading to the fact that the induced-fit and conformational selection mechanism should be regarded as the two opposing extreme scenarios for binding [91]–[95]. The extent to which the two binding scenarios contribute to the overall binding mechanism is dependent on multiple conditions, including the interactions between the associated biomolecules [89], the range of the interactions [89], the concentrations of the biomolecules [96] and the rate of the conformational transition [97]. It is worth noting that DPO4-DNA recognition is quite different from the common “mixed” binding model, in which the induced-fit is often followed by selecting the favored conformations in that [94], [98], [99]. Before the final binding, DNA-binding “selects” DPO4 in the A-state instead of the I-state, which is found to be an inevitable intermediate along the conformational change from the A- to B-state. At the last binding stage, the inactive states of DPO4 are induced by coupled binding and folding to evolve to the B-state. This rate-limiting binding step at moderate salt concentrations follows typical “induced-fit” mechanism. We argue that the novel binding mechanism is due to the entropy caused by the geometry of the DNA, which favors A- or B-state as the binding proceeds from the above discussions. This binding mechanism proposed here can be applied into the case when the geometries of the associated biomolecules are so significant that the entropy has a dramatic effect in the binding.

### The general and unique characteristics for Y-family DNA polymerases binding to DNA

The multi-step DNA binding mechanism involving conformational changes in the orientation of the LF domain relative to the polymerase core due to the flexible linker described here for DPO4 may be shared by other Y-family DNA polymerases as well. For example, crystal structures of human DNA polymerases *κ* (hPOL*κ*) show an open to closed conformational change of up to 50 *Å* in the position of the LF domain relative to the polymerase core between the apo and DNA bound states [29], [30], similar to the A- to B-state change in DPO4. Yeast DNA polymerase eta (yPOL*η*) may also undergo a similar conformational change during DNA binding [23], [31], [32]. However, crystal structures suggest that the change is likely more modest with only an 8° rotation of the LF domain relative to the polymerase core of yPOL*η* [23], [31], [32]. Interestingly, the LF domain of hPOL*κ* was observed in two different conformations in the apo state [29], [30], both of which are distinct from the conformation observed in DNA bound state, which is quite consistent with our simulation results that show a conformational equilibrium primarily between the A-state and I-state in the US which are distinct from the B-state which dominates in the BS.

During replication *in vivo*, DNA polymerases are believed to be regulated by sliding clamps (PCNA in archaea and eukaryotes or the *β* clamp in bacteria) which coordinate proteins at a DNA replication fork [100]–[102]. Structural studies have shown that in the DPO4-PCNA complex, DPO4 adopts an extended conformation in which the position of the LF domain relative to the F, T and P domains is different from that in both the Apo- and DNA-bound DPO4 structures [42]. This extended conformation of DPO4 in which the LF domain does not form interactions with the F and T domain is quite similar to the I-state observed in our simulations. In addition, the experimental prediction that the flexible linker allows for the existence of equilibrium between multiple conformations of DPO4 with and without a PCNA or DNA binding partner is also confirmed by our simulations. It is worth noting that the results here seem to be specific to the Y-family polymerases, since other families of DNA polymerases do not have a LF domain and thus lack a conformational change between the A- and B-states. The unique structural features of the flexible linker and the LF domain [103], which lead to a complex multi-step binding process involving multiple conformational states, may serve to regulate Y-family DNA polymerases to function feasibly and efficiently when the replicative polymerases stalls at a damage site during the DNA replication process.

In our work, we developed a two-basin SBM for DPO4 binding to its target DNA with explicit consideration of the specific interactions between protein and DNA. Our results showed that DPO4 undergoes non-specific 3D diffusion, then a non-specific short-range adjustment sliding on DNA and finally specific binding with conformational changes, which occur in all of the recognition stages during DNA binding. The efficiency of the recognition is modulated by salt concentrations, the flexibility of the linker and the conformational dynamics in DPO4. Our results provide a clear illustration of how a protein finds its target sites on DNA with conformational changes under different conditions. In addition to the unique insights we have provided into the mechanism of DPO4-DNA binding, the methods we have developed here will provide a powerful tool for future investigations of the binding pathways of many different proteins and their targeted DNA substrates.

## Materials and Methods

**Figure 9:**
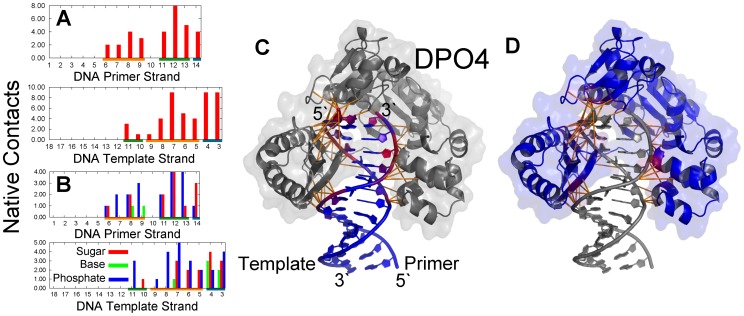
Native contacts between DPO4 and DNA in native bound structure. (A) Native contacts for each nucleotide in DNA. DPO4 interacts with DNA in native bound structure at three different regions, which are colored orange, olive green and dark cyan, corresponding to the major groove, minor groove and the terminal of the DNA duplex, respectively. (B) Native contacts for the sugar, base and phosphate group in DNA. (C, D) The native structure of DPO4-DNA binary complex. In (C), the sugar, base and phosphate groups in DNA are colored from blue to red, corresponding to contact number from 0 to 5; while DPO4 is colored grey. In (D), the residues in DPO4 are colored from blue to red, corresponding to the DPO4-DNA contact number from 0 to 5, while DNA is colored grey. The DPO4-DNA contacts are drawn by orange lines in (C) and (D).

The plain SBM has been widely used for investigating the protein folding [45], [46] and binding [47]–[49]. Here we adapted the plain SBM with unique native structure to the two-basin SBM to explore the conformational dynamics in DPO4 as well as the binding dynamics to DNA. Our model is constructed on a coarse-grained level. Each residue in DPO4 is represented by a 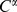 atom and each nucleotide in DNA representing by three beads, located at the centroid of the sugar, base and phosphate groups without heterogeneity. Only Arg and Lys in DPO4 are modeled to carry one positive charge and Asp and Glu in DPO4, as well as phosphate pseudo atoms in DNA are modeled to carry one negative charge. The native contact map of DPO4 is a mixture of the native contacts in the A- and B-state native structure. The regions with large-scale changes in angle and dihedral between the A- and B-state are defined as hinges. The hinges are expected to be more flexible than the other regions in DPO4. In our system, the hinges of DPO4 correspond to the disordered region in the F domain when it is in the A-state and the flexible linker connecting the T and LF domain. In general, DPO4 is initially coordinated by sliding clamps (PCNA in archaea and eukaryotes or the *β* clamp in bacteria) before binding to DNA during translesion synthesis. After that, DPO4 only has to search for the replication fork within a small stretch of DNA. Therefore, a short length of primer/template 14/16-mer (5′–*GGGACCCTTCGAAT*–3′/5′–*TTATTC–GAAGGGTCCC*–3′) DNA substrate is used in our simulations to describe DPO4-DNA recognition. In reality, different lengths of DNA require proteins to spend different times sliding on DNA, resulting in different binding kinetics. The effect can be measured by quantifying the interplay between non-specific and specific binding in protein-DNA recognition [104]. In DPO4-DNA binary complex (PDB: 2RDJ), DPO4 contacts with DNA through abundant interactions, including the LF domain interacting with the major groove of the DNA duplex and the T domain interacting with the minor groove, as well as the interactions between DPO4 and the terminal nucleotides on the DNA (Figure 9). These interactions are used to build the specific protein-DNA contact map. Notably, DNA is kept rigid and frozen in space while DPO4 is set to be free. The rigidity of DNA in our model reduces the flexibility of the protein-DNA interfaces, which may promote the rapid and efficient finding of the target site [19]. However, the whole recognition process will not be significantly changed due to the short length of DNA here. Further investigations on DNA dynamics participating into protein-DNA recognition need to improve the coarse-grained model taking into account flexibility of DNA [105]–[107]. The two-basin SBM here is built according to the crystal structures of apo-DPO4 (PDB: 2RDI) and DPO4 in DPO4-DNA binary complex (PDB: 2RDJ), based on our previous work [50], [51]. Therefore the Hamiltonian can be expressed by:
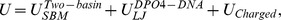
where 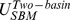 is the two-basin Hamiltonian for SBM, 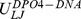 is the potential for specific contacts between DPO4 and DNA, *U_Charged_* is the potential of electrostatic interactions, which exist between all pairs of oppositely charged beads. The long-range non-electrostatic interactions are represented by LJ potential while the electrostatic interactions are described by Debye-Hückel model, in which the effect of salt concentration is modulated by the length of Debye radius [108]. Recently, coarse-grained models with and without specific protein-nucleotide contacts were widely applied into the investigation of protein-DNA recognition mechanism and the results were consistent with the experiments [3], [6], [18], [75], [105], [109]–[112].

All the simulations were performed using Gromacs 4.0.5 [113], integrated by Langevin equation with constant friction coefficient 1.0 *ps* ^−1^. All the bonds were constrained by LINCS algorithm to ensure the MD step of 2 *fs* [114]. To achieve a better sampling, we used REMD [55] to explore the thermodynamics. The neighbor replica attempted to exchange with each other at every 5000 MD steps. The average of acceptance exchanged ratios in our REMD simulations is from 17% to 37%, leading to sufficient sampling. After REMD simulations, all the trajectories were collected and free energy landscapes are calculated by Weighted Histogram Analysis Method (WHAM) algorithm [115].

For kinetics, 200 constant temperature simulations started from different dissociative A-state of DPO4 and DNA with different velocities for each condition were performed. Different conditions in our kinetic simulations refers to different salt concentrations, different flexibility of linker and different conformational dynamics in DPO4. In practice, these can be realized by modulating the length of Debye radius, the strength of the angle and dihedral potential related to the linker and the strength of the specific contacts in the A- or B-state. Details can be found in Text S1 and our previous work [50], [51].

## Supporting Information

Text S1 Models and simulation details and additional results.
(PDF)
