# Supplementary material for: Dynamic Conformational Change Regulates the Protein-DNA Recognition: An Investigation on Binding of a Y-Family Polymerase to Its Target DNA
Source: PLoS Comput Biol. 2014 Sep 4;10(9):e1003804. doi: 10.1371/journal.pcbi.1003804 (PMC4154647; doi:10.1371/journal.pcbi.1003804)
Supplement: Text S1 — Models and simulation details and additional results. (PDF) [file pcbi.1003804.s001.pdf]

Text S1

1 Models and simulation details

1.1 Two-basin structure-based model

Protein folding energy landscape has been found to be minimally frustrated funnel with biasing to the unique native structure to solve Levinthal's paradox [1–6]. Structure-based model (SBM), which only takes into consideration the interactions in native structure, has removed the energetic roughness to guarantee the principle of minimal frustrations [7, 8]. SBM has been widely applied into protein folding, biomolecular binding with global conformational changes. The simulation findings obtained from SBM are consistent with experiments in many aspects [9–14]. Furthermore, SBM simulations can provide more detailed dynamics at molecular level than experimental measurements.

The biomolecules are in dynamics, the conformational changes may occur when the biomolecules perform their function [15]. Even without interacting with others, the biomolecules can transform from one state to the other state in favor of realizing function. Therefore, there may be a number of energy minima rather than only one single energy basin located at the bottom of the energy funnel to ensure the functional dynamics [16–21]. To describe the conformational dynamics, the plain single-basin SBM has to be extended to multiple-basin SBM to capture the multiple-minima at the bottom of the energy landscapes [22–26].

In our study, the two-basin SBM was built to explore the conformational dynamics of DPO4 during binding to the target site in DNA. Based on our previous work on exploring the conformational dynamics in maltose-binding protein (MBP) and adenylate kinase (ADK) [25, 26], we constructed the two-basin Hamiltonian using the native contacts formed in Apo state (PDB: 2RDI) and DNA-Bound state (PDB: 2RDJ). For DPO4, there are four charged residues: Arg and Lys carry one positive charge while Asp and Glu carry one negative charge. For DNA, we used another coarse-grained representation. Each nucleic acid in DNA is represented by three beads, standing for the phosphate, sugar and base groups, respectively. All the three beads are located at the center of mass of the corresponding groups and each phosphate pseudo atom takes one negative charge [27–35]. DNA was kept frozen during the simulations and there are only inter-chain interactions in DNA. The coarse-grained Hamiltonian is expressed by:

$$\begin{aligned} U &= U_{SBM}^{Two-basin} + U_{LJ}^{DPO4-DNA} + U_{Charged} \\ &= U_{Bonds} + U_{Angles} + U_{Dihedrals} + U_{LJ} + U_{Charged} \end{aligned}$$

, where $U_{Bonds} = k_b(r - r_0)^2$. r is the distance of pseudo bond between adjacent C^{α} and with the subscript 0, it corresponds to r in the A- or B-state. The strength of the bond term is controlled by k_b. The expression of pseudo angle and pseudo dihedral are divided into non-hinge and hinge regions by comparing the differences of the angle and dihedral degree in the A- and B-state as shown in Figure S1.

$$U_{Angles} + U_{Dihedral} = U_{Non-hinge} + \epsilon_{Hinge} U_{Hinge}$$

, where $U_{Non-hinge}$ and U_{Hinge} have same functional form as:

$$k_{\theta}(\theta - \theta_0)^2 + k_{\phi}[(1 - cos(\phi - \phi_0)) + 0.5(1 - cos3(\phi - \phi_0))]$$

, where θ and ϕ are the pseudo angle and pseudo dihedral and with the subscript 0, they correspond to θ and ϕ in the A- or B-state. The strengths are controlled by k_{θ} and k_{ϕ}, respectively. The hinge region indicates that it will undergo large angle and dihedral changes during the transition and is supposed to be more flexible than the non-hinge region. ϵ_{Hinge} is set to control the flexibility of the hinge. The smaller ϵ_{Hinge} is, the more flexible hinge will be. It is worth noting that in our model, we used the B-state of DPO4 as the template to build the potential of pseudo bond terms as well as the pseudo angle

and pseudo dihedral terms for non-hinge regions. Alternatively, we can use the A-state of DPO4 as the template, but the results are robust for the choices [22, 25, 26].

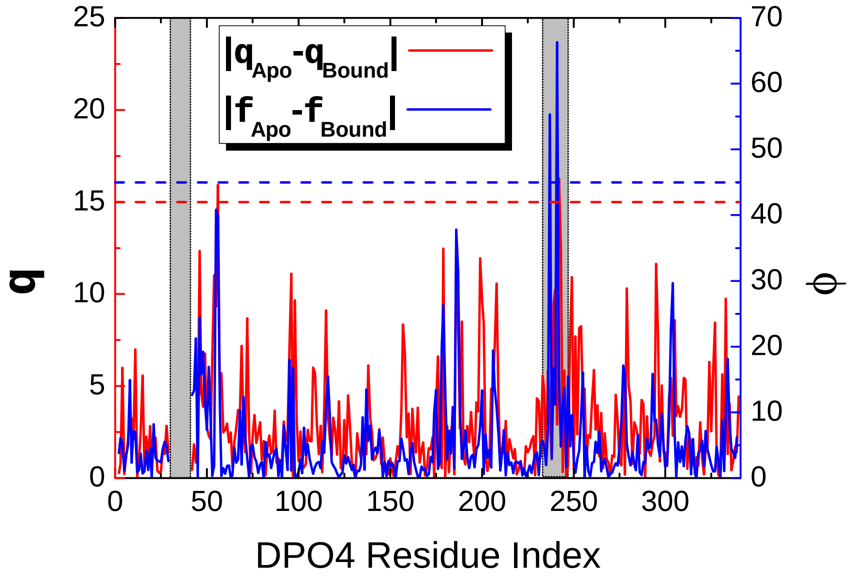

Figure S1. The regions of hinge are determined by the differences of angle. The angle differences of pseudo angle and pseudo dihedral between the A- and B-state of DPO4 are shown. The angle is in the unit of degree (°). The threshold values for angle bending and dihedral rotation are set to 15° and 45°, respectively. Notice that for residue 33-40 in the F domain, they are very flexible without specific structure in the A-state and this region therefore can be regarded as a hinge region. The other hinge region are residues 234-244, corresponding to the flexible linker between the T and LF domain.

The Lennard-Jones (LJ) interactions in our model are divided into native contacts of intra-chain terms in DPO4 and inter-chain terms between DPO4 and DNA, as well as the non-native contacts of the volume repulsive terms.

$$U_{LJ} = U_{LJ}^{DPO4} + U_{LJ}^{DPO4-DNA} + U_{Repulsive}$$

To build the native contacts in U_{LJ}^{DPO4}, we calculated the distances of the native contacts in the A- and B-state of DPO4 (Figure S2). The native contact map is built by Contacts of Structural Units (CSU) software [36]. As shown in Figure S2 and Figure S3, the native contacts are divided into: (1) “Core” contacts, which are similar in distance in the A- and B-state of DPO4, corresponding to the native contacts shared by the A- and B-state of DPO4; (2) specific “Apo” contacts, which are all located at the interface between the LF and T domain, corresponding to the specific inter-domain contacts of the LF domain in the A-state of DPO4; (3) specific “iBound” contacts, which are all located at the interface between the LF and F domain, corresponding to the specific inter-domain contacts of the LF domain in the B-state of DPO4; (4) specific “fBound” contacts, which are all related to folding of the hinge region in the F domain, corresponding to the specific intra-domain contacts of the F domain in the B-state of DPO4; (5) “discarded” contacts, which are a little different in distance between the A- and B-state of DPO4 and can be discarded in the Hamiltonian, according to the previous investigations [22, 26, 37–39].

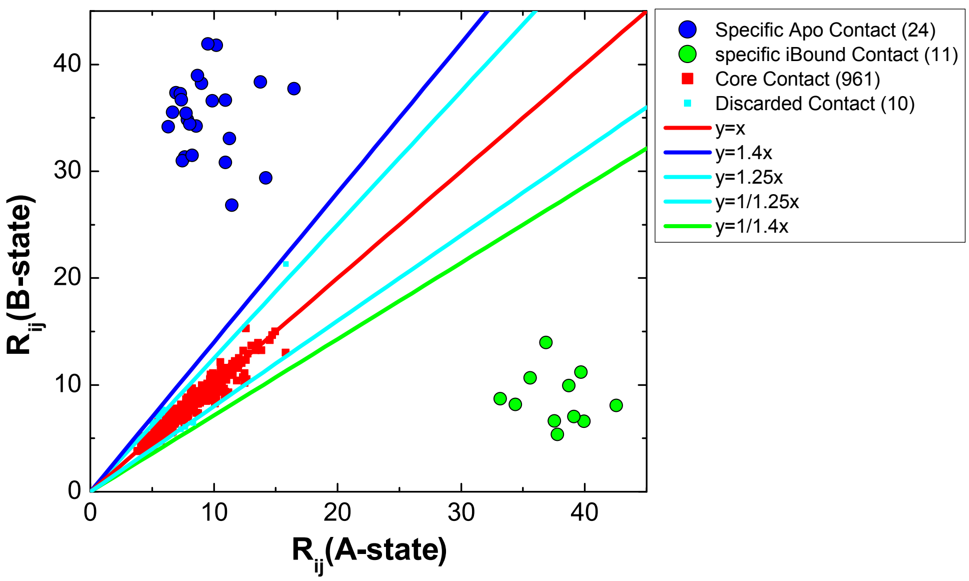

Figure S2. The differences of native contact distances between the A- and B-state of DPO4. Notice that the number of "fBound" contacts, which correspond to the folding native contacts of disordered loop in the F domain in the B-state of DPO4, is 14. R_{ij} is the distance of native contact of residue i and residue j and is in the unit of $\mathring{A}$.

Therefore, the expression of U_{LJ}^{DPO4} is:

$$
\begin{aligned}
& U_{LJ}^{DPO4} \\
& = U_{LJ}(Core) + U_{LJ}(Apo) + U_{LJ}(iBound) + U_{LJ}(fBound) \\
& = \sum_{Parts} \epsilon_{Parts} U_{LJ}
\end{aligned}
$$

, where U_{LJ} is the Lennard-Jones potential:

$$U_{LJ} = \epsilon_{LJ}[5(\frac{R_{ij}}{r_{ij}})^{12} - 6(\frac{R_{ij}}{r_{ij}})^{10}]$$

R_{ij} is distance of the native contact (i, j) in native structure and r_{ij} is the distance of the native contact (i, j) in trajectories during the simulations. The strength of LJ potential is controlled by ϵ_{LJ}. The strengths of different groups of native contacts are modulated by ϵ_{Parts}, where the subscript "Parts" can be "Core", "A", "iB" and "fB", corresponding to "Core", "Apo", "iBound" and "fBound", respectively. The specific native contacts in the A- and B-state are supposed to be weaker than the shared contacts, namely the core contacts, due to the fact that the conformational dynamics happens by only switching the specific-state contacts rather than significantly changing the core contacts, leading to global conformational changes. Therefore the ratio of the strength of the specific contacts to the strength of the core contact equaling 0.5 was applied throughout our simulations. It is worth noting that for "fBound" contacts, which monitor folding of the flexible hinge in the F domain, the strength of these contacts should be weaker than the "Apo" and "iBound" contacts, since this region is disordered in the A-state. The strategy that the contacts for inter-domain and inter-domain adopt different interaction strengths was widely used in molecular simulations, especially in modeling of Intrinsically Disordered Proteins' (IDPs') binding-folding [40, 41].

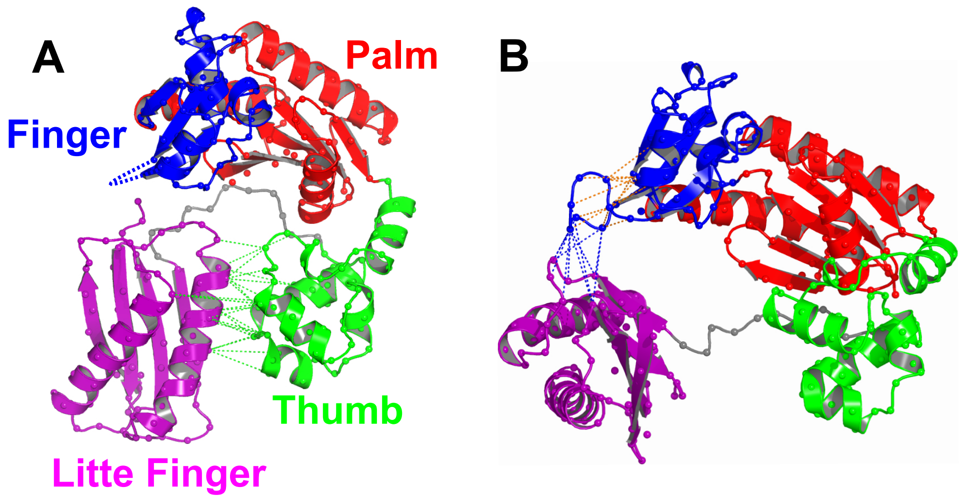

Figure S3. The structural differences of DPO4 and the native contact map in the (A) A-state and (B) B-state of DPO4. DPO4 has a typical polymerase core consisting of a palm (P), finger (F) and thumb (T) in addition to a fourth domain as little finger (LF) domain with a flexible linker. The specific "Apo" contacts are all located at the interface between the LF domain and T domain, and are colored green. The specific "iBound" contacts are all located at the interface between the LF domain and F domain, are colored blue. The specific "fBound" contacts correspond the intra-domain interactions related to folding of disordered regions in the F domain in the A-state, and are colored orange. The P, F, T and LF domain in DPO4 are colored red, blue, green and purple, respectively. The flexible linker between the T and LF domain are colored grey. Notice that DNA is not shown in the B-state for a better visualization.

The DPO4-DNA Lennard-Jones interactions can be expressed by:

$$U_{LJ}^{DPO4-DNA} = \epsilon_{iDNA} U_{LJ}$$

The native contact map between DPO4 and DNA is constructed by cut-off algorithm: if the distance between any heavy atoms in DPO4 and DNA is smaller than 4 $\AA$, there is a inter-chain native contact between DPO4 and DNA [30]. Therefore, the DPO4-DNA binding is specific recognition, attracted by the specific contacts at the interface. ϵ_{iDNA} is used to modulate the strength of the native contacts between DPO4 and DNA.

By investigating the contact between DPO4 and DNA (Figure S4), we found that: for DNA primer strand, the major groove, corresponding to the nucleotide of base number 6-9, entirely interacts with the LF domain; the minor groove, corresponding to the nucleotide of base number 11-13, mostly interacts with the T domain (except one sugar of nucleotide with base number 13 located at the edge of the minor groove, interacting with the P domain). While for DNA template strand, the major groove, corresponding to the nucleotide of base number 5-9, mostly interacts with the LF domain and the linker between the LF and T domain (except one sugar of the nucleotide with base number 5, interacting with the F domain and one sugar of the nucleotide with base number 6, interacting with the P domain); the minor groove, corresponding to the nucleotide of base number 10-11, entirely interacts with the T domain. In addition, the edge of the major groove in template strand, corresponding to the nucleotide of base number 3-4, interacts with the LF domain and F domain (mostly in or around the disordered region); the edge of the minor groove in primer strand, corresponding to the nucleotide of base number 1, entirely interacts with the P domain. To sum up, the major groove of the DNA, mostly interacts with the LF domain and linker while the minor groove of DNA, mostly interacts with the T domain. Besides, there are many specific contacts between the terminal of the DNA with the LF, P and F domain, respectively. The phenomenon

that the primary binding site on the DNA duplex is usually located at the major groove [42, 43], is also found by our analysis that there are less contacts at the minor groove.

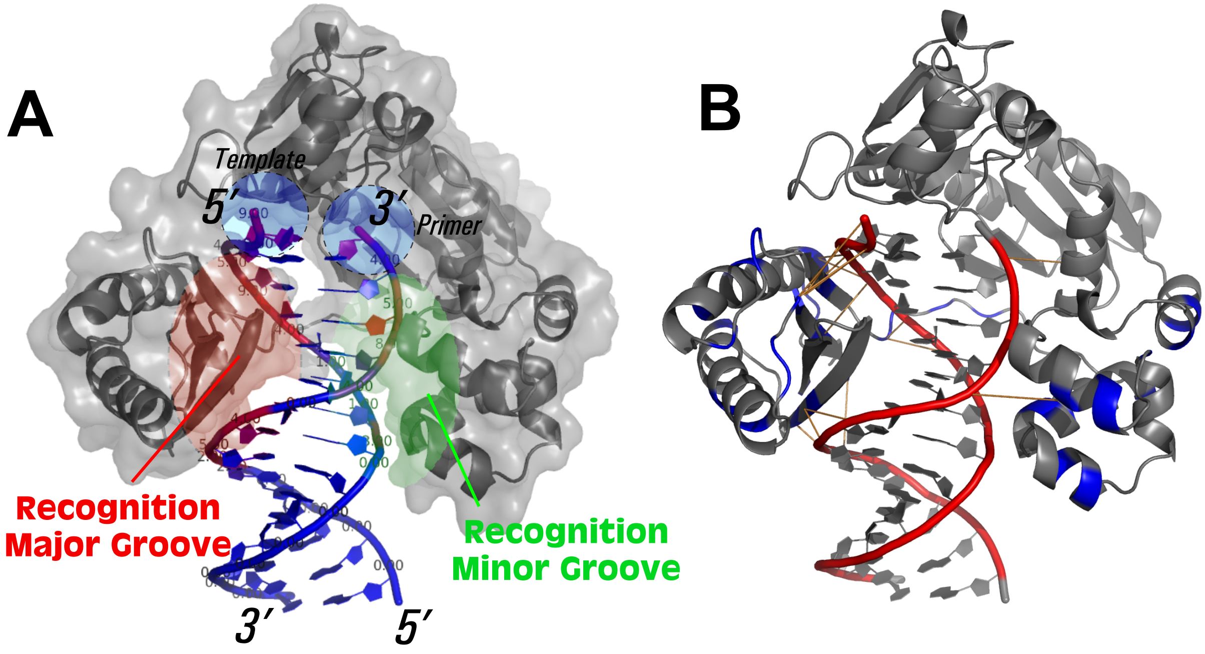

Figure S4. Native contacts between DPO4 and DNA in native DPO4-DNA binary structure. (A) Structural illustration of DPO4-DNA binary complex. The residues in DPO4 as well as sugar, base and phosphate groups in DNA are colored from blue to red, corresponding to the native contact number between DPO4 and DNA from 0 to 5. There are three regions in DNA that form the native contacts with DPO4 in native bound structure: recognition major groove, recognition minor groove and the terminal of DNA. (B) The native electrostatic contact map in native bound structure are shown. The positively charged residues (Lys and Arg) in our model at the T, LF domain and linker in DPO4 are colored blue. The phosphate groups in DNA, which are negatively charged in our model, are colored red. The charged residue-nucleotide contacts are plotted by orange lines.

The non-native interactions in our models are represented as the volume repulsive forces with the expression:

$$U_{Repulsive} = \epsilon_{NC}(\frac{\sigma_{NC}}{r_{ij}})^{12}$$

Considering the ion screening effect in electrostatic interactions, the charged interactions are described by Debye − Hückel model:

$$U_{Charged} = K_{coulomb}B(k)\frac{q_i q_j exp(-kr_{ij})}{\epsilon_r r_{ij}}$$

$B(\kappa)$ is the salt-dependent coefficient; q_i is the charge of the pseudo atom i. κ^{-1} is the Debye screening length which is directly affected by salt concentration; ϵ_r is dielectric constant and was set to 80 throughout the simulations. So the relationship between κ and salt concentration C_{salt} can be written explicitly: $\kappa \approx 3.2\sqrt{C_{salt}}$. The exact physical meaning of $K_{coulomb}, \kappa, B(\kappa), q_i, q_j$ can be found here [28]. In our simulations, we changed the strength of the electrostatic interactions by modulating the salt concentrations.

In our work, the parameters $k_r = 10000.0\ kJ \cdot mol^{-1} \cdot nm^{-2}, k_\theta = 20.0\ kJ \cdot mol^{-1}, K_\phi = 1.0\ kJ \cdot mol^{-1}, \epsilon_{LJ} = 1.0\ kJ \cdot mol^{-1}, \epsilon_{NC} = 1.0\ kJ \cdot mol^{-1}, \sigma_{NC} = 4.0\ \AA, K_{coulomb} = 138.94\ kJ \cdot mol^{-1} \cdot nm \cdot e^{-2}$ are used throughout our studies. Notice that if the native contacts between the oppositely charged atoms no matter they are in DPO4 or at the interface between DPO4 and DNA, forms salt bridge, the corresponding ϵ_{LJ} will be scaled to 0.1 $kJ \cdot mol^{-1}$ to achieve a similar energy contributions of the other native contacts [27]. For REMD simulations, we set the strength of the "Core" contact $\epsilon_{Core} = 2.0$, while the strength of specific "Apo", "iBound" and "fBound" contacts were $\epsilon_A = 1.0$, $\epsilon_{iB} = 1.0$ and $\epsilon_{fB} = 0.5$, respectively. We also increased the flexibility of the hinge to be $\epsilon_{Hinge} = 0.01$. The parameters were also tested in the kinetic simulations to see their effects in kinetic simulations.

We used fraction of native contacts Q as the order parameter to monitor the DNA binding process with conformational changes. Due to the small number of native contacts formed in specific "Apo", "iBound", "fBound" contact pairs, we used a continuous function instead of discrete counting number to calculate Q:

$$Q = \sum_{i,j} \frac{1 - ((r_{ij} - R_0)/R_{ij})^{10}}{1 - ((r_{ij} - R_0)/R_{ij})^{20}}$$

, where $R_0 = 0.3R_{ij}$.

To investigate the non-native contacts, we introduced a cut-off algorithm based on our previous investigation [44]. We used a dual-radius criterion to calculate the contacts [44]. If the distance between the C^α atom in DPO4 and the pseudo atom in DNA is shorter than 5 $\AA$, we set the contact value as 1, while if the distance is between 5 $\AA$ and 8 $\AA$, we set contact value as 0.5. We applied the cut-off algorithm to calculate contact in native DPO4-DNA complex and found the contact value is 91, a little higher than 79, which is the number of native contacts.

1.2 Temperature effect

In order to see the effect of temperature on the process of DNA recognition, we plotted 2D free energy landscapes (Figure S5).

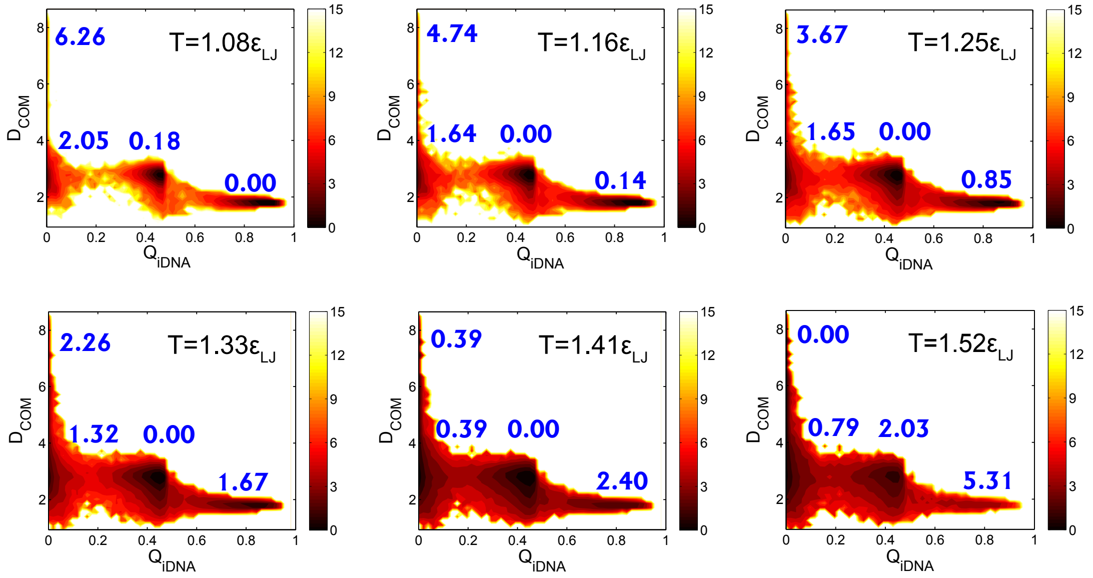

Figure S5. The free energy landscapes as a function of Q_{iDNA} and D_{COM} at different temperatures. The blue numbers around the free energy minima are the corresponding free energy values of each stage. D_{COM} is in the unit of nm and free energy is in the unit of kT.

1.3 Simulation protocols

All the simulations were preformed with Gromacs 4.0.5 [45]. The topology files for Gromacs were generated by the SMOG@ctbp webserver (http://smog-sever.org) with modifications [46]. The coarse grained simulations used Langevin equation with constant friction coefficient 1.0 ps^{-1}. All the bonds were constrained by LINCS algorithm [47] to ensure a longer time step of 2 fs. The trajectories were saved at every 1 ps. DPO4 and DNA were placed in a cubic box and a harmonic potential was added if the two chains were farther than 8.0 nm, resulting in an effective concentration of DPO4: 0.8 mM. During the simulations, DNA was kept frozen in the space without conformational changes while DPO4 was set to be free.

For thermodynamic simulations, a series of 32 parallel REMD for a certain case were performed. Each replica in REMD ran for 2×10^8 MD steps and attempts to exchange with the neighbor at every 5000 MD steps. The average of acceptance exchanged ratios for the REMD were found to be from 17% to 37%, leading to sufficient data sampling. After the REMD simulations, the data at all temperatures were collected by Weighted Histogram Analysis Method (WHAM) algorithm [48] to calculate the free energy landscapes at the specified temperature.

For kinetic simulations, a series of 200 constant temperature MD started from different configures at unbound states which consist of the A-state of DPO4 and DNA, and different velocities for a certain case were performed. The transition time and transition number between the ajacent stages, which were indicated from the free energy landscapes in thermodynamic results, were collected, then the Mean Passage Time (MPT) was calculated. During the kinetic simulations, there may be some trajectories which can not reach the final stage and the corresponding data is missing. This is similar to the survival life testing experiments, in which the event of interest (death) may be prevented for some of the items of the sample (loss). In common practice, the mean survival life time is estimated by changing the single experimental time to the death time if it is censored [49]. The single experimental time can be the largest observation time or the largest event time. Here, we used the largest observation time to represent the MPT if the trajectory is censored.

References

1. Levinthal C (1969) Proceedings in mossbauer spectroscopy in biological systems. In: Mossbauer Spectroscopy in Biological Systems: Proceedings of a meeting held at Allerton House, Monticello, Illinois. pp. 22-24.

2. Bryngelson JD, Wolynes PG (1987) Spin-glasses and the statistical-mechanics of protein folding. Proc Natl Acad Sci USA 84: 7524-7528.

3. Bryngelson JD, Wolynes PG (1989) Intermediates and barrier crossing in a random energy-model (with applications to protein folding). J Phys Chem 93: 6902-6915.

4. Leopold PE, Montal M, Onuchic JN (1992) Protein folding funnels - a kinetic approach to the sequence structure relationship. Proc Natl Acad Sci USA 89: 8721-8725.

5. Karplus M (1997) The levinthal paradox: yesterday and today. Fold Des 2: S69-S75.

6. Dill KA, Chan HS (1997) From levinthal to pathways to funnels. Nat Struct Biol 4: 10-19.

7. Bryngelson JD, Onuchic JN, Socci ND, Wolynes PG (1995) Funnels, pathways, and the energy landscape of protein-folding - a synthesis. Proteins 21: 167-195.

8. Go N (1983) Theoretical studies of protein folding. Annu Rev Biophys Bioeng 12: 183–210.

9. Clementi C, Nymeyer H, Onuchic JN (2000) Topological and energetic factors: What determines the structural details of the transition state ensemble and ”en-route” intermediates for protein folding? an investigation for small globular proteins. J Mol Biol 298: 937-953.

10. Levy Y, Wolynes PG, Onuchic J (2004) Protein topology determines binding mechanism. Proc Natl Acad Sci USA 101: 511-516.

11. Levy Y, Cho SS, Onuchic J, Wolynes PG (2005) A survey of flexible protein binding mechanisms and their transition states using native topology based energy landscapes. J Mol Biol 346: 1121-1145.

12. Levy Y, Onuchic JN (2006) Mechanisms of protein assembly: Lessons from minimalist models. Acc Chem Res 39: 135-142.

13. Chu X, Wang Y, Gan L, Bai Y, Han W, et al. (2012) Importance of electrostatic interactions in the association of intrinsically disordered histone chaperone chz1 and histone h2a.z-h2b. PLoS Comput Biol 8: e1002608.

14. Chu X, Gan L, Wang E, Wang J (2013) Quantifying the topography of the intrinsic energy landscape of flexible biomolecular recognition. Proc Natl Acad Sci USA 110: E2342-E2351.

15. Whitford PC, Sanbonmatsu KY, Onuchic JN (2012) Biomolecular dynamics: order–disorder transitions and energy landscapes. Rep Prog Phys 75: 076601.

16. Frauenfelder H, Sligar SG, Wolynes PG (1991) The energy landscapes and motions of proteins. Science 254: 1598-1603.

17. Tsai CJ, Kumar S, Ma BY, Nussinov R (1999) Folding funnels, binding funnels, and protein function. Protein Sci 8: 1181-1190.

18. Tsai CJ, Ma BY, Nussinov R (1999) Folding and binding cascades: Shifts in energy landscapes. Proc Natl Acad Sci USA 96: 9970-9972.

19. Kumar S, Ma BY, Tsai CJ, Sinha N, Nussinov R (2000) Folding and binding cascades: Dynamic landscapes and population shifts. Protein Sci 9: 10-19.

20. Papoian GA, Wolynes PG (2003) The physics and bioinformatics of binding and folding - an energy landscape perspective. Biopolymers 68: 333-349.

21. Schug A, Onuchic JN (2010) From protein folding to protein function and biomolecular binding by energy landscape theory. Curr Opin Pharmacol 10: 709-714.

22. Whitford PC, Miyashita O, Levy Y, Onuchic JN (2007) Conformational transitions of adenylate kinase: Switching by cracking. J Mol Biol 366: 1661–1671.

23. Lu Q, Wang J (2008) Single molecule conformational dynamics of adenylate kinase: energy landscape, structural correlations, and transition state ensembles. J Am Chem Soc 130: 4772–4783.

24. Okazaki Ki, Takada S (2008) Dynamic energy landscape view of coupled binding and protein conformational change: induced-fit versus population-shift mechanisms. Proc Natl Acad Sci USA 105: 11182–11187.

25. Wang Y, Tang C, Wang E, Wang J (2012) Exploration of multi-state conformational dynamics and underlying global functional landscape of maltose binding protein. PLoS Comput Biol 8: e1002471.

26. Wang Y, Gan L, Wang E, Wang J (2012) Exploring the dynamic functional landscape of adenylate kinase modulated by substrates. J Chem Theory Comput 9: 84–95.

27. Levy Y, Onuchic JN, Wolynes PG (2007) Fly-casting in protein-dna binding: Frustration between protein folding and electrostatics facilitates target recognition. J Am Chem Soc 129: 738-739.

28. Azia A, Levy Y (2009) Nonnative electrostatic interactions can modulate protein folding: Molecular dynamics with a grain of salt. J Mol Biol 393: 527-542.

29. Givaty O, Levy Y (2009) Protein sliding along dna: Dynamics and structural characterization. J Mol Biol 385: 1087-1097.

30. Toth-Petroczy A, Simon I, Fuxreiter M, Levy Y (2009) Disordered tails of homeodomains facilitate dna recognition by providing a trade-off between folding and specific binding. J Am Chem Soc 131: 15084-15085.

31. Marcovitz A, Levy Y (2009) Arc-repressor dimerization on dna: folding rate enhancement by colocalization. Biophys J 96: 4212–4220.

32. Vuzman D, Azia A, Levy Y (2010) Searching dna via a ”monkey bar” mechanism: The significance of disordered tails. J Mol Biol 396: 674-684.

33. Vuzman D, Levy Y (2010) Dna search efficiency is modulated by charge composition and distribution in the intrinsically disordered tail. Proc Natl Acad Sci USA 107: 21004–21009.

34. Vuzman D, Polonsky M, Levy Y (2010) Facilitated dna search by multidomain transcription factors: cross talk via a flexible linker. Biophys J 99: 1202–1211.

35. Vuzman D, Levy Y (2012) Intrinsically disordered regions as affinity tuners in protein–dna interactions. Mol Biosyst 8: 47–57.

36. Sobolev V, Sorokine A, Prilusky J, Abola EE, Edelman M (1999) Automated analysis of interatomic contacts in proteins. Bioinformatics 15: 327-332.

37. Levy Y, Cho SS, Shen T, Onuchic JN, Wolynes PG (2005) Symmetry and frustration in protein energy landscapes: A near degeneracy resolves the rop dimer-folding mystery. Proc Natl Acad Sci USA 102: 2373–2378.

38. Schug A, Whitford PC, Levy Y, Onuchic JN (2007) Mutations as trapdoors to two competing native conformations of the rop-dimer. Proc Natl Acad Sci U S A 104: 17674–17679.

39. Baxter EL, Jennings PA, Onuchic JN (2011) Interdomain communication revealed in the diabetes drug target mitoneet. Proc Natl Acad Sci USA 108: 5266–5271.

40. Turjanski AG, Gutkind JS, Best RB, Hummer G (2008) Binding-induced folding of a natively unstructured transcription factor. PLoS Comput Biol 4: e1000060.

41. Ganguly D, Chen J (2011) Topology-based modeling of intrinsically disordered proteins: Balancing intrinsic folding and intermolecular interactions. Proteins Struct Funct Bioinform 79: 1251–1266.

42. Benos PV, Lapedes AS, Stormo GD (2002) Is there a code for protein–dna recognition? probab (ilistical) ly. Bioessays 24: 466–475.

43. Rohs R, Jin X, West SM, Joshi R, Honig B, et al. (2010) Origins of specificity in protein-dna recognition. Annu Rev Biochem 79: 233.

44. Wang J, Wang Y, Chu X, Hagen SJ, Han W, et al. (2011) Multi-scaled explorations of binding-induced folding of intrinsically disordered protein inhibitor ia3 to its target enzyme. PLoS Comput Biol 7: e1001118.

45. Hess B, Kutzner C, van der Spoel D, Lindahl E (2008) Gromacs 4: Algorithms for highly efficient, load-balanced, and scalable molecular simulation. J Chem Theory Comput 4: 435-447.

46. Noel JK, Whitford PC, Sanbonmatsu KY, Onuchic JN (2010) Smog@ctbp: simplified deployment of structure-based models in gromacs. Nucleic Acids Res 38: W657–W661.

47. Hess B, Bekker H, Berendsen HJ, Fraaije JG (1997) Lincs: a linear constraint solver for molecular simulations. J Comput Chem 18: 1463–1472.

48. Kumar S, Bouzida D, Swendsen RH, Kollman PA, Rosenberg JM (1992) The weighted histogram analysis method for free-energy calculations on biomolecules .1. the method. J Comput Chem 13: 1011-1021.

49. Efron B (1967) The two sample problem with censored data. In: Proceedings of the fifth Berkeley symposium on mathematical statistics and probability. University of California Press, Berkeley, volume 4, pp. 831-853.

2 Results

Table S1. Protein-DNA interaction energy in each stage for the A-, I- and B-state of DPO4 at $T = 1.25\epsilon_{LJ}$.

**US**	**A-state**		**I-state**		**B-state**	
Energy Part *	Native	Non-native	Native	Non-native	Native	Non-native
E_{Elect}	0.00 ± 0.03	-0.08 ± 0.54	-0.01 ± 0.04	-0.18 ± 0.82	-0.01 ± 0.02	-0.04 ± 0.08
E_{LJ}	0.00 ± 0.02	0.01 ± 0.12	-0.00 ± 0.01	0.01 ± 0.09	0.00 ± 0.00	0.00 ± 0.00
**EC**	**A-state**		**I-state**		**B-state**	
Energy Part	Native	Non-native	Native	Non-native	Native	Non-native
E_{Elect}	-0.57 ± 0.53	-15.03 ± 4.18	-0.63 ± 0.72	-15.28 ± 4.74	-0.26 ± 0.13	-16.88 ± 1.78
E_{LJ}	-0.29 ± 0.55	1.25 ± 1.29	-0.32 ± 0.61	1.40 ± 1.44	-0.02 ± 0.01	1.19 ± 0.67
**IS**	**A-state**		**I-state**		**B-state**	
Energy Part	Native	Non-native	Native	Non-native	Native	Non-native
E_{Elect}	-10.69 ± 0.81	-11.10 ± 1.04	-10.63 ± 0.84	-11.47 ± 1.38	-10.12 ± 1.12	-11.61 ± 1.40
E_{LJ}	-18.42 ± 2.57	0.21 ± 0.24	-18.44 ± 2.54	0.33 ± 0.49	-18.50 ± 2.15	0.40 ± 0.51
**BS**	**A-state** ♯		**I-state**		**B-state**	
Energy Part	Native	Non-native	Native	Non-native	Native	Non-native
E_{Elect}			-11.00 ± 0.00	-10.14 ± 0.00	-10.42 ± 0.80	-12.26 ± 1.18
E_{LJ}			-35.95 ± 0.00	0.42 ± 0.00	-42.26 ± 4.37	0.43 ± 0.38

(*) Energy is in the unit of ϵ_{LJ}.
(♯) The A-state of DPO4 cannot be observed in BS during our simulations at this temperature.

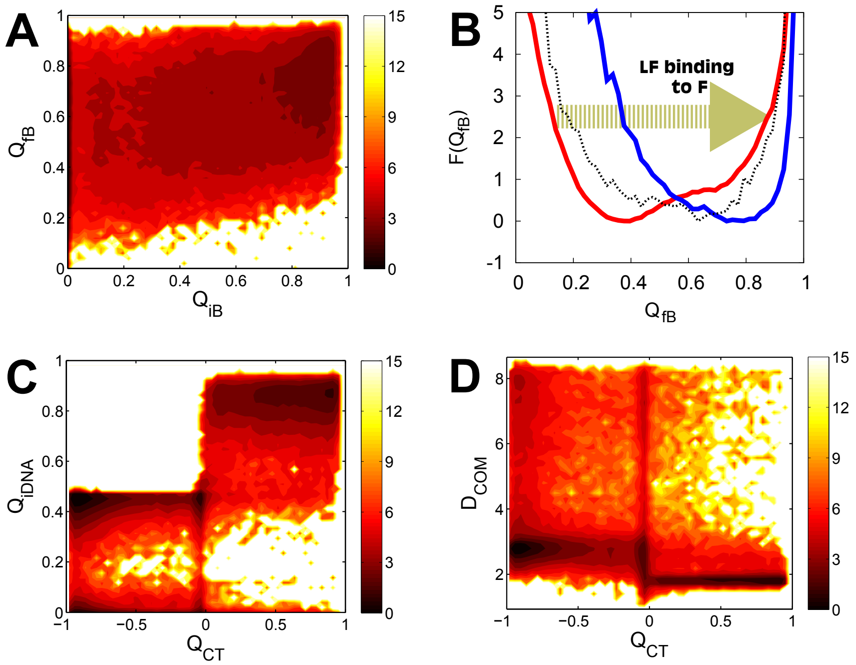

Figure S6. Free energy landscapes of DPO4 binding to DNA. (A) The 2D free energy is shown as a function of Q_{iB} and Q_{fB}. Q_{iB} and Q_{fB} are both related to the formation of specific native contacts in the B-state of DPO4. Q_{iB} monitors the native inter-domain binding contacts between the LF and F domain, while Q_{fB} monitors the native intra-domain folding contacts of the disordered loop of the F domain in the A-state of DPO4. (B) The 1D free energy is shown as a function of Q_{fB}. Red and blue lines are the free energy at the cases of the LF domain unbinding and binding to the F domain, corresponding to the state with $Q_{iB} = 0$ and $Q_{iB} > 0.8$, respectively. The dotted line is the free energy at the transition state of the LF domain binding to the F domain, corresponding to $0 < Q_{iB} < 0.1$. (C) The 2D free energy is shown as a function of Q_{CT} and Q_{iDNA}. Q_{CT}, defined as $Q_{iB} - Q_A$, can monitor the conformational transitions of DPO4. Q_{iDNA} is the fraction of native contacts formed by the interface of DPO4 and DNA. (D) The free energy are shown as a function of Q_{CT} and D_{COM}. D_{COM} is the center of mass between DPO4 and DNA. D_{COM} is in the unit of nm. Free energy is plotted at $T = 1.25\epsilon_{LJ}$ and is in the unit of kT.

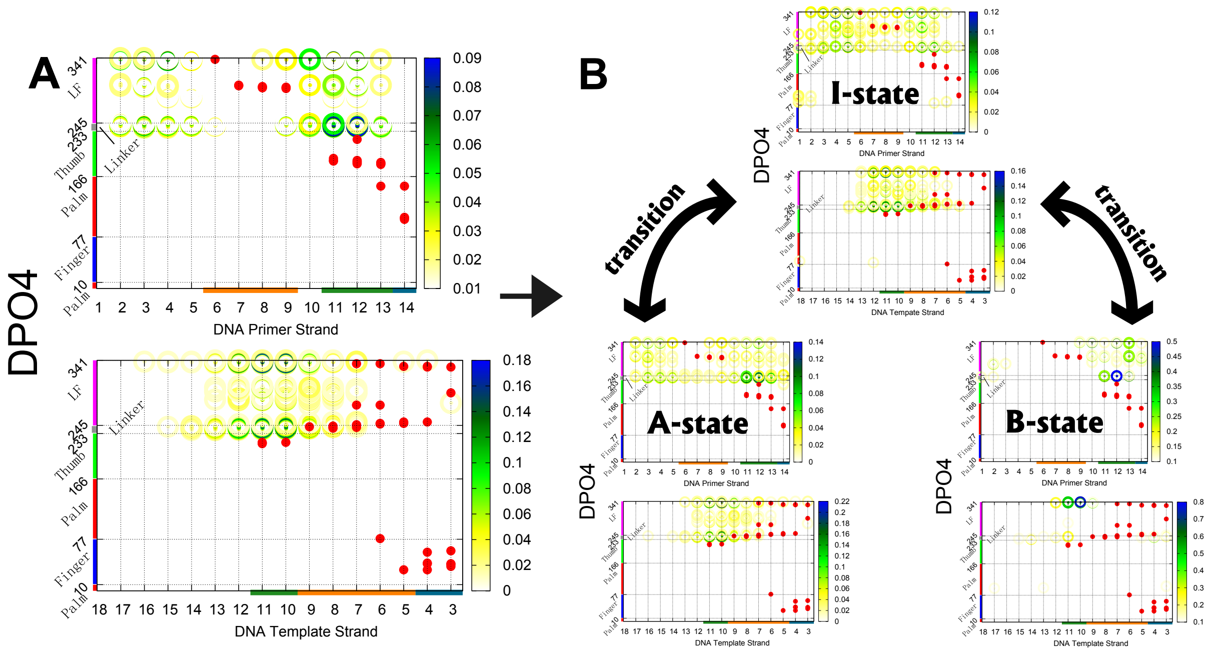

Figure S7. The binding contact maps between DPO4 and DNA in the EC for three different states of DPO4. (A) The whole average of the three different states of the contact maps between DPO4 and DNA in the EC. (B) The binding contact maps between DPO4 and DNA in the EC for the three different states of DPO4. The number of the contacts are drawn by colored circles and the contacts formed in native DPO4-DNA binary structure are drawn by red points. Notice that DPO4 in the B-state can form stronger binding contacts with DNA than it in the A- and I-state. Nevertheless, the contacts of DPO4 in the B-state contribute little to the whole average contact binding due to its very small population.

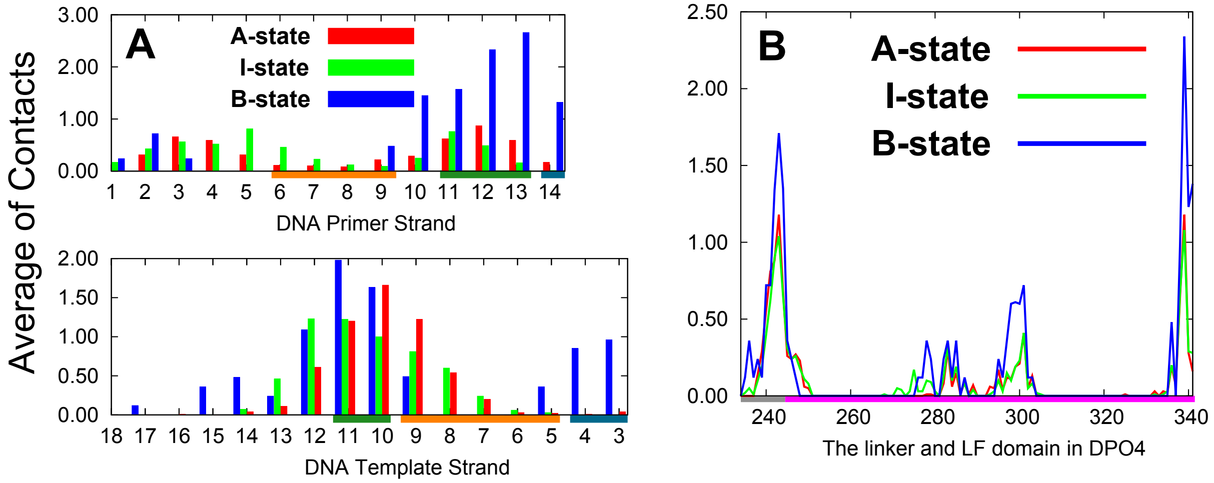

Figure S8. The binding contacts between DPO4 and DNA in the EC for the three different states of DPO4. (A) Average of contacts of DNA are shown by primer and template strand, separately. (B) Average of contacts of DPO4 are shown. Notice that DNA can form stronger contacts when DPO4 is in the B-state than DPO4 is in the A- and I-state. Nevertheless, the contacts of DPO4 in the B-state contribute little to the whole average contact binding due to its very small population.

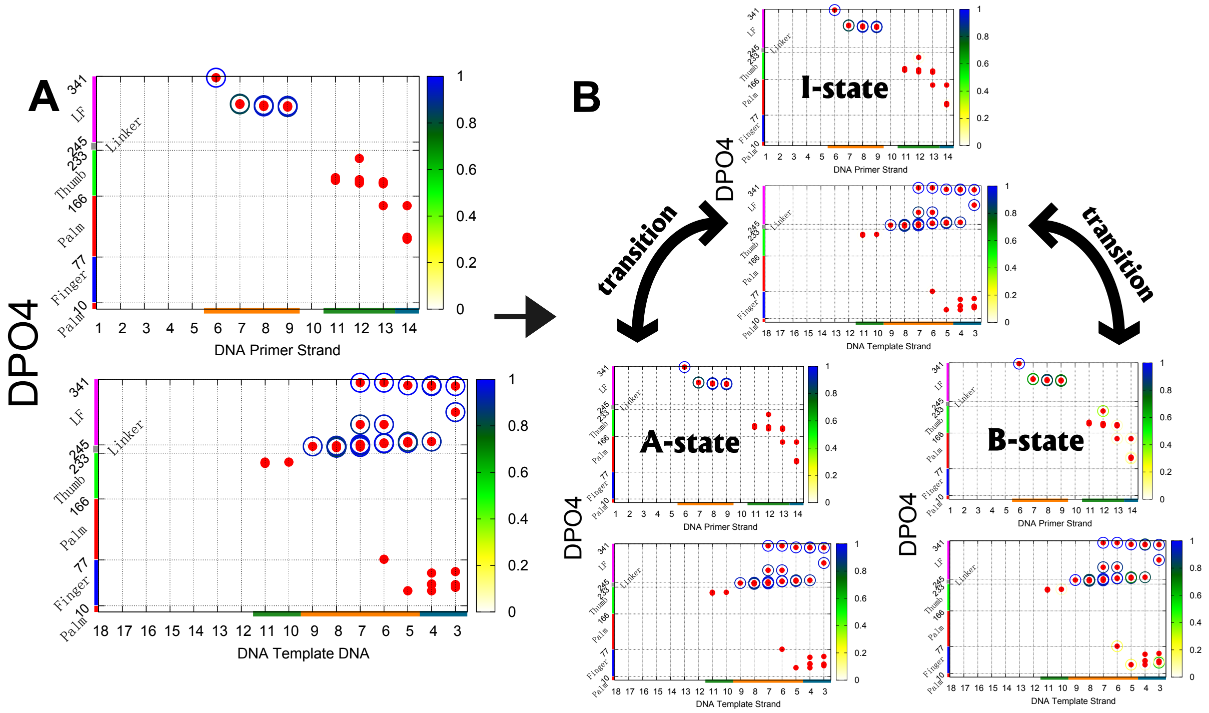

Figure S9. The native binding contact map between DPO4 and DNA in the IS. (A) The whole average of the three different states of the native contact maps between DPO4 and DNA in the IS. (B) The native binding contact maps between DPO4 and DNA in the IS for the three different states of DPO4. The probability of native contacts are drawn by colored circles and the contacts formed in native DPO4-DNA binary structure are drawn by red points. Notice that DPO4 in the three states share very similar binding native contact map.

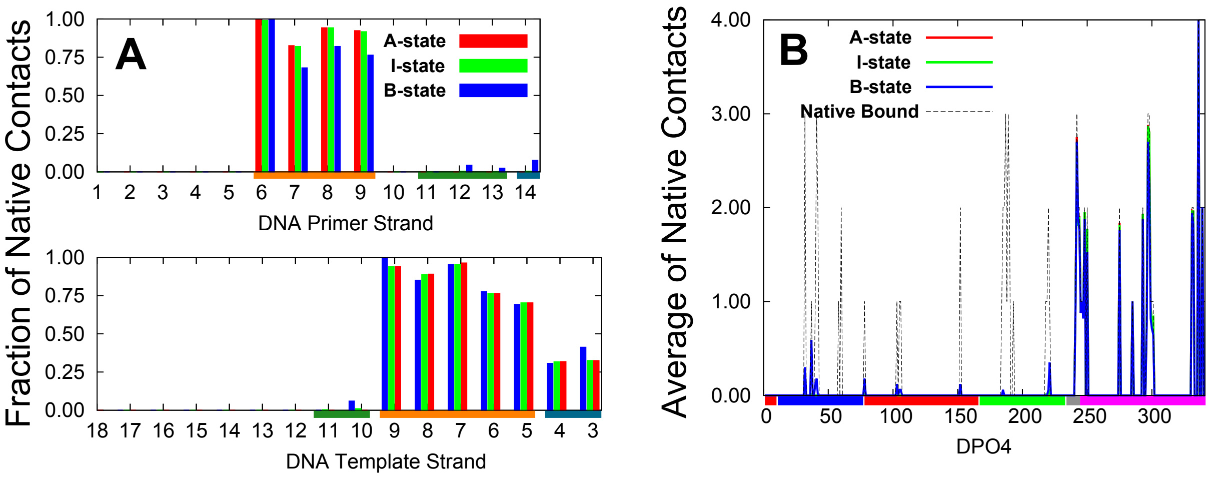

Figure S10. The native binding contacts between DPO4 and DNA in the IS for the three different states of DPO4. (A) Fraction of the native contacts of DNA are shown by primer and template strand, separately. (B) Average of native binding contacts of DPO4. Notice that DPO4 in the three states share very similar binding native contacts.
